# Supplementary material for: Optical neural network via loose neuron array and functional learning
Source: Nat Commun. 2023 May 3;14:2535. doi: 10.1038/s41467-023-37390-3 (PMC10156674; doi:10.1038/s41467-023-37390-3)
Supplement: Supplementary file 1 — Supplementary Document [file 41467_2023_37390_MOESM1_ESM.pdf]

Supplementary Material for Optical Neural Network via Loose Neuron Array and Functional Learning

Yuchi Huo[1,2,3], Yifan Peng[4], Chen Gao[2], Wei Hua[2], Qing Yang[2], Haifeng Li[2], Hujun Bao[2,1], Rui Wang[1], and Sung-eui Yoon[3]

[1]State Key Lab of CAD&CG, Zhejiang University, Hangzhou, 310000, China
[2]Zhejiang Lab, Hangzhou, 310000, China
[3]Korea Advanced Institute of Science and Technology, Deajeon 34141, South Korea
[4]The University of Hong Kong, Hong Kong SAR, China

ABSTRACT

This document provides additional information and figures to support the paper "Optical Neural Network via Loose Neuron Array and Functional Learning".

S1 Loose Neuron Array

The artificial neural network concept inspires a physical counterpart, loose neuron array, enabling the transform from handcrafted designs to non-handcrafted designs. It is interesting that merely connecting many artificial neurons outperforms sophisticated handcrafted algorithms in a large number of tasks. While connecting artificial neurons is very handy on computers, there is no trivial way to connect and train arbitrary physical neurons because the nonideal design, material, structure, manufacture, fabrication, and run-time environment hinder gradient calculation. On the other hand, enabling the connection and training of arbitrary physical neurons releases us from these burdens and reveals new possibilities for hardware designing, chip manufacturing, and system control.

S1.1 Numerical Simulation

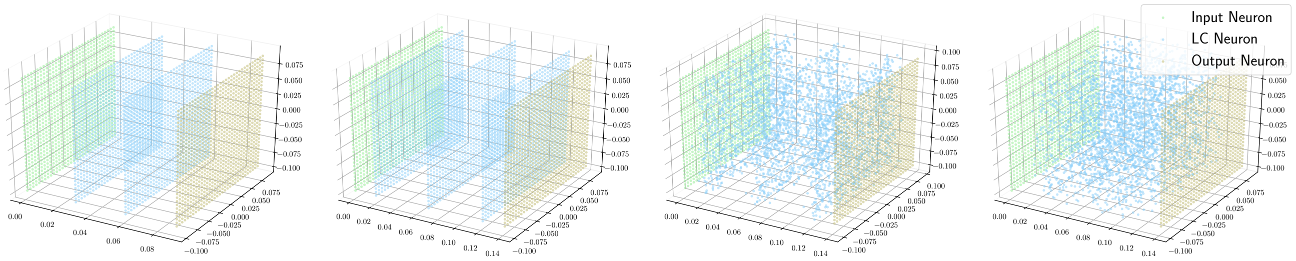

(a) Regular-2 Array **(b)** Regular-3 Array **(c)** Normal-3 Array **(d)** Uniform Array

	Regular-2	Regular-3	Normal-3	Uniform
**LC Neurons**	2048×3	3072×3	3072×3	3072×3
**1-Layer MNIST**	91.03%	92.07%	92.40%	92.45%
**2-Layer MNIST**	96.61%	97.30%	97.55%	97.65%
**3-Layer CIFAR10**	47.48%	50.61%	51.73%	52.53%

Figure S1. Accuracy of using FL to train different neuron arrays with the numerical simulation.

Here we verify this concept with numerical simulation (Figure S1), where the input neurons are point light sources with 70 degree field of view, the output neurons are energy gatherers, and the LC neurons are small components that can attenuate incoherent light passing through them. All neurons are modeled as 6 mm length cubes. While simulating the incoherent light signal, the solid angles, distances, and occlusions between neurons are carefully considered to simulate the light propagation. There are 1024×3 input and output neurons, respectively, forming an ONN that can process 32×32 pixels RGB images. The simulated arrays are regarded as black boxes except the

input and LC neurons are writable, and the output neurons are readable. We use FL to train such non-handcrafted neuron arrays for classification tasks without knowing the configuration of neurons. Figure S1 shows the test results.

The regular-2 array has two layers of regular LC neuron grid between the input and output neurons. Each layer consists of 1024×3 LC neurons. We physically realize and test this array through our LFNN prototype (Section S2). However, the actual LFNN prototype neurons are not perfectly aligned and consistent, as the regular-2 array. As shown in Section S2, the actual LFNN prototype is not calibrated and suffers from unpredictable noises, inference, and reflectance. The actual LFNN prototype achieves similar accuracy to that of the regular-2 array simulation (Section S4), which demonstrates the robustness of our FL paradigm in real-world systems.

The regular-3 array has 3072×3 regularly aligned LC neurons in three layers. It is an enhanced version of the regular-2 array. Compared to the regular-2 array, the regular-3 array shows higher accuracy, implicating that it is possible to further increase the LFNN's performance by merely adding more neurons. We conduct an additional experiment to evaluate the impact of spacing between layers (Table S1). The numerical simulation results verify an intuition that both the number of neurons and the spacing can impact the prediction accuracy. Increasing the number of neurons between the input and output layers can increase the computing density and optimization space of the system. Increasing the spacing between layers makes each neuron can modulate signal within a larger scattering angles, which also help increasing the optimization space. However, we find that while increasing the space from 60 mm to 120 mm has evident improvement, increasing the space from 120 mm to 240 mm has only marginal impact.

The normal-3 array also has 3072×3 LC neurons. We add a normal-distribution disturbance to each LC neuron to imitate a nonperfect regular-3 array with fabrication bias. Specifically, we first draw 3 random numbers from a normal distribution of standard deviation 0.25 to distribute each neuron within a unit cube with 99.99% confidence. Then the cube is resized to match the actual physical size of the whole system, where the two axes on the LC plane are scaled to pixel width and the last axis is scaled to one-third of the spacing between two LC layers. In such a case, the overlap between the neurons of the same LC plane is maximized. For the offset between two LC layers, we make the offset relatively larger to simulate misalignments. Because the LC neurons' exact locations are unknown, we capture their light spots on the output plane to calculate their distances to the output plane, then use k-means to cluster the LC neurons into 10 layers to build the FNN. Compared to the regular-3 array, the LC neurons of normal-3 array have irregular impact factors but still yield better results, thanks to the data-driven FL paradigm. While handcrafted designs contain inevitable assumptions on the actual physics, non-handcrafted designs create inhomogeneous physics that can be exploited by the FL paradigm in a data-driven way. For example, some light paths between the input and output planes have relatively higher modulation resolutions due to LC neurons' inhomogeneous distribution, which are leaned and used to propagate relatively important information. An intuitive analogy is that randomly initializing artificial neural networks gets better results compared to normalized initialization.

The uniform array is the utmost generalization of loose neuron array, where all 3072×3 LC neurons are uniformly distributed between the first and the last LC layers. For each neuron, we draw a random number from a uniform distribution to determine its distance to the first and the last LC layer while keeping the other two axes regularly aligned as the regular-3 array. It simulates the cases of spreading neurons within liquid or airspace. We also cluster these LC neurons into 10 layers.

Interestingly, the most general and non-handcrafted configuration, the uniform array, achieves the best performance. This observation confirms our initial conjecture that non-handcrafted designs can replace and even work better than handcrafted designs. The non-handcrafted design not only releases us from many realization difficulties, but also allows new possibilities in hardware design, chip manufacturing, and system control.

Table S1. Accuracy comparison of different layer spacing with the numerical simulation. All neuron arrays are regularly aligned with two or three LC layers, where the spacing between the input and output layers are reported in the first row. The LC layers equally split the spacing. For example, the distance between two LC layers is 30 mm for the regular-3 array of 120 mm spacing.

	**Regular-2**	**Regular-2**	**Regular-2**	**Regular-3**	**Regular-3**	**Regular-3**
**Spacing**	60 mm	120 mm	240 mm	60 mm	120 mm	240 mm
**LC Neurons**	2048×3	2048×3	2048×3	3072×3	3072×3	3072×3
**1-Layer MNIST**	85.88%	91.70%	91.75%	88.89%	91.73%	92.00%
**2-Layer MNIST**	96.30%	97.24%	97.25%	97.01%	97.51%	97.79%
**3-Layer CIFAR10**	45.65%	47.34%	47.99%	46.68%	49.42%	50.02%

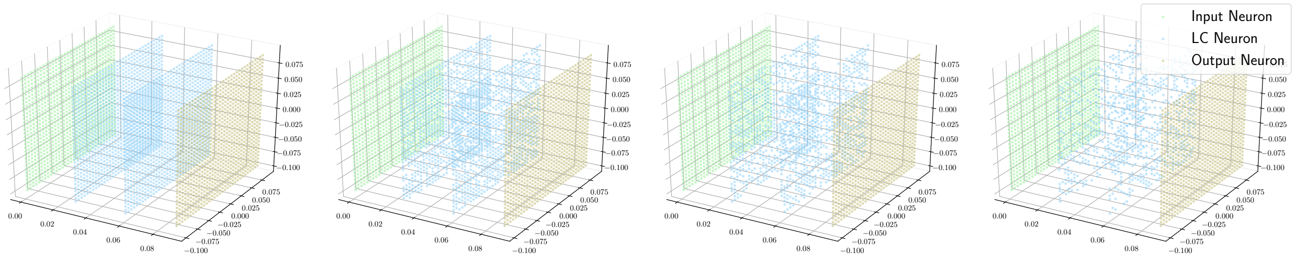

(a) Bernoulli-0 Array **(b)** Bernoulli-20 Array **(c)** Bernoulli-40 Array **(d)** Bernoulli-60 Array

	**Bernoulli-0**	**Bernoulli-20**	**Bernoulli-40**	**Bernoulli-60**
**LC Neurons**	2048×3	1638×3	1229×3	819×3
**Simulation**	89.80%	82.07%	79.17%	77.29%
**LFNN**	89.13%	81.83%	79.36%	75.65%

Figure S2. Neuron arrays with 0%, 20%, 40% and 60% randomly malfunctioning neurons. We report the accuracy of testing different Bernoulli arrays on both the numerical simulation and the actual LFNN device for the 1-layer MNIST classification.

S1.2 Physical Assessment

We further conduct a test to verify that training random neurons is also feasible in real-world systems. We apply different Bernoulli distributions to the regular-2 array, numerically and physically, to generate random neuron arrays Figure S2. We randomly select and deactivate certain ratios of LC neurons in our LFNN prototype. In addition, *all* input and output gains are deactivated to emphasize the change of LC neuron number.

The test results are summarized in Figure S2. The physically captured LFNN output is comparable to that of the equal-configuration simulation, confirming that it is possible to train random physical neurons in practice. The experiment also assesses the robustness of training loose neuron arrays with the FL paradigm by showing reasonable accuracy even with up to 60% of malfunctioning neurons. Given that robustness, there can be many possible configurations of loose neuron arrays to adapt to different actual environments and applications.

S2 Light Field Neural Network

We physically prototype a loose neuron array, termed light field neural network (LFNN), to realize the regular-2 array and Bernoulli array by randomly deactivating neurons (Section S1). We use off-the-shelf components, e.g., liquid crystal display (LCD) panels, polarizers, and a machine vision camera, without tedious calibration. As a result, the actual LFNN is not precisely the same as the regular-2 array.

S2.1 Hardware Implementation

The applied LCD panels are expected to show a high transmittance and good linearity between the applied voltage and the polarization rotation angle. Among available LCD panels, we use Chimei Innolux AT070TN83 as the optical layer. The photograph of our prototype is shown in Figure S3. A modified backlight system adapted from a commercial projector is used to illuminate the input plane. The front and rear polarizing films are removed from the front of two LCDs. A diffuser and a polarizing film are located at the camera's focal plane as the output plane. All layers were assembled into acrylic frames separately, and all frames are installed on the optical table. The distance setting between the layers is 30 mm. Here we first set the distance between the input plane and the output plane as 90 mm so that the central neuron's energy distribution can roughly cover the entire output plane (Figure S4). Then we equally split the spacing to insert two LC layers. The output plane images were acquired by a machine vision camera (The Imaging Source DFK 33G274) with the pixel pitch of 4.40 μm, the resolution of $1,600 \times 1,200$, and the bit depth 12 bit. The focal length and F-number of the camera are 12.5 mm and 1.6, respectively. Because the device is not fine-tuned, we use the pixels in these planes' center areas as neurons. Specifically, 15×13 pixels in the input plane, liquid crystal 1 (LC1), and liquid crystal 2 (LC2) are jointly controlled as one input neuron and LC neurons. 12×15 pixels in the camera's image plane are read together as one output neuron.

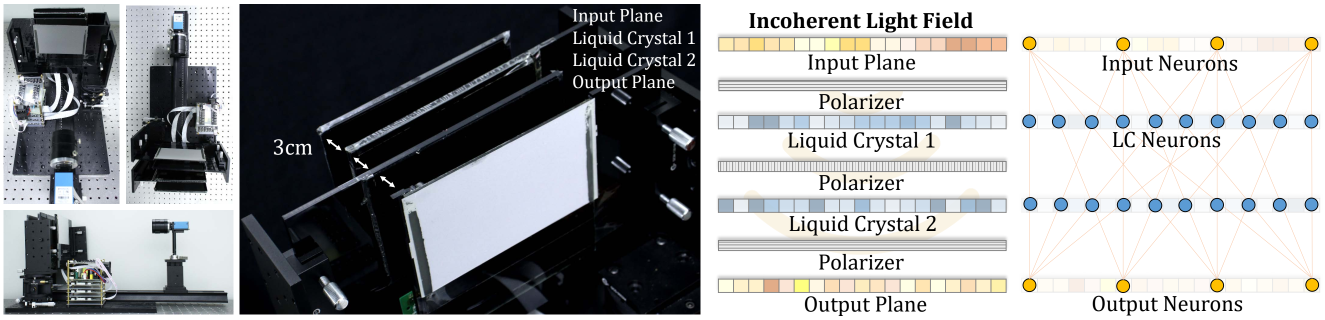

Figure S3. Light field neural network. The LFNN prototype consists of an input plane, an output plane, two layers of liquid crystal, and three perpendicular linear polarizers. The output plane is a scattering plane followed by a camera to acquire the data. We use an extra LCD as the input plane by representing artificial neurons with pixels.

S2.2 Optical Characterization

Note that we use only off-the-shelf, low-cost components to build the LFNN prototype without fine-tuning, thereby the neurons are not consistent, and the connections are uncertain. The whole system embodies a neuron array with many loosely connected physical neurons that are non-differentiable and beyond the description of an explicit mathematical model. It is not easy to explicitly characterize such an optical system's light property with numerical values, so we resort to the visualization of the energy distribution under different hardware parameters.

Figure S4 characterizes the input plane's neuron by capturing each neuron's energy distribution on the output plane with the neuron parameters of LC1 and LC2 all set to 1. The classic point spreading function (PSF) model indicates that each neuron should exhibit a circular spot-type energy distribution on the output plane. However, the practical hardware neurons create unpredictable irregularities while the light travels through many micro-structures and materials. For example, there are varying interference rings, irregular light spots, random patterns, structured shadows, and noise. To our best knowledge, there is no method to robustly train the parameters or calculate the gradients of such a to date.

Figure S5 and Figure S6 visualize the characterized optical behavior of LC1. For Figure S5, we set the parameter of the input plane's central neuron as 1, but others' as 0 and set all neuron parameters as 1 for LC2, then set only one of LC1's neuron parameter to 1 in sequence to capture a slice of the light field. The input plane's central neuron is only affected by a small part of the LC1's neurons. We can observe different patterns for different color channels.

For Figure S6, all neural parameters of the input plane are set to 1 to yield less noisy captured patterns. However, the captured patterns are still not symmetrical circles or regular distributions.

Figure S7 and Figure S8 are the counterparts for LC2. LC2 has a wider influence area with respect to the input plane's central neuron. In Figure S7, there are strange red patterns diverged from the primary bright spots, which have irregular and asymmetric patterns for unknown reasons.

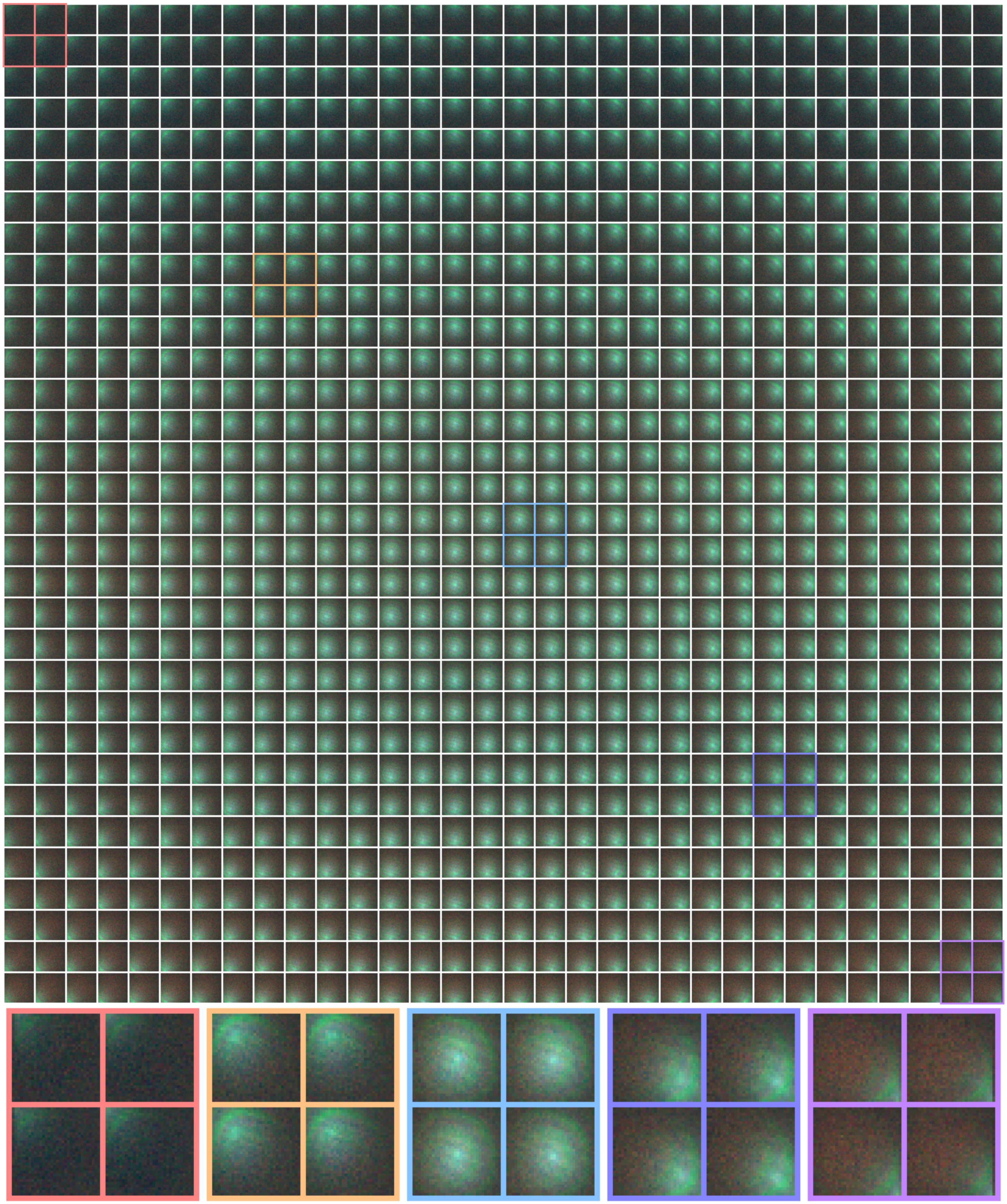

Figure S4. Captured energy distributions of input plane's neurons. To capture this light field slice, we set one neuron parameter as 1 and others' as 0 in sequence for the input plane; set all neuron parameters as 1 for LC1; and set all neuron parameters as 1 for LC2. The captured outputs are linearly normalized between 0 and 1 for visualization.

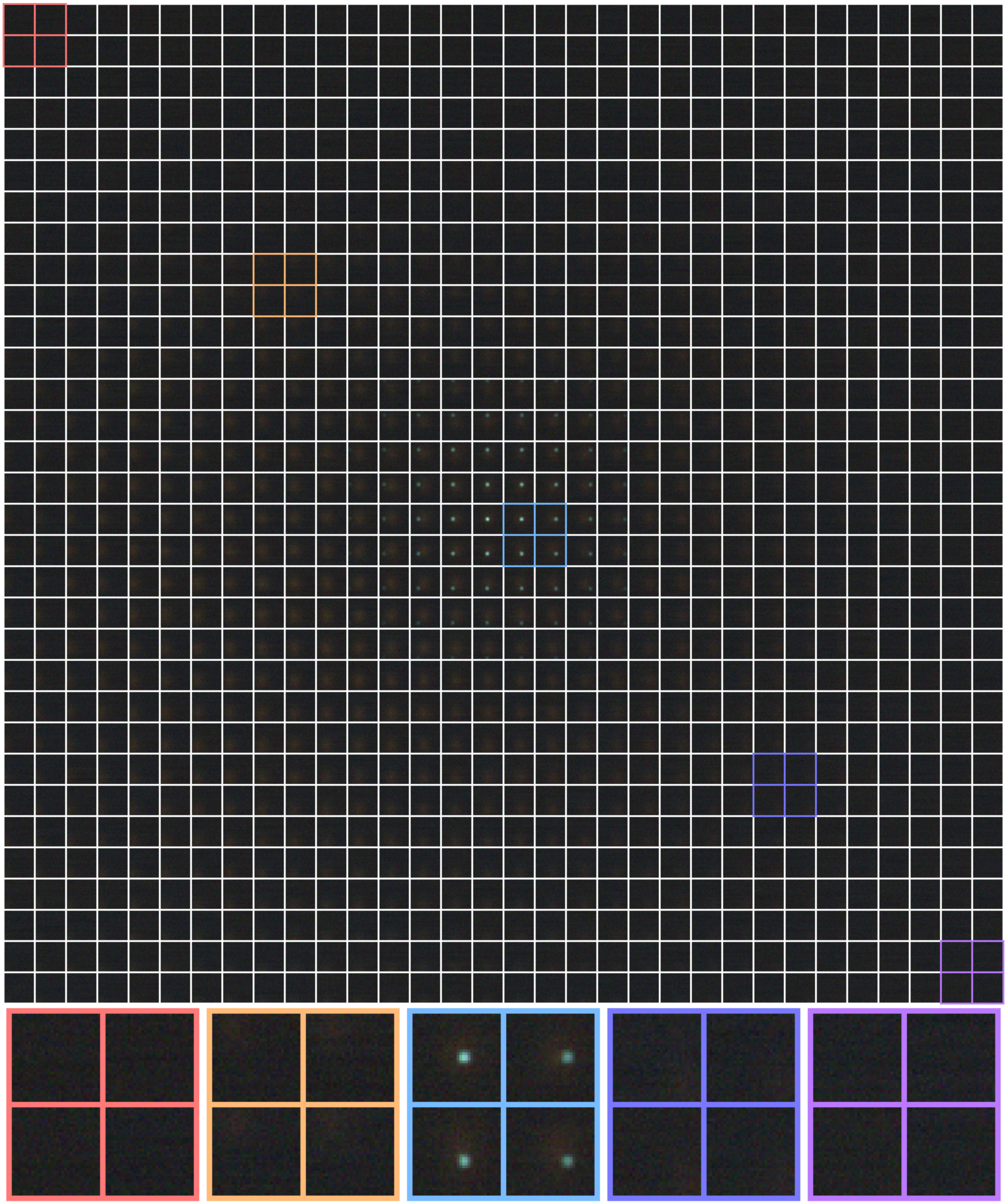

Figure S5. Captured energy distributions of the input plane's central neuron with respect to LC1. To capture this light field slice, we set one neuron parameter as 1 and others' as 0 in sequence for LC1; set the central neuron's parameter as 1 but others' as 0 for the input plane; and set all neuron parameters as 1 for LC2. The captured outputs are linearly normalized between 0 and 1 for visualization.

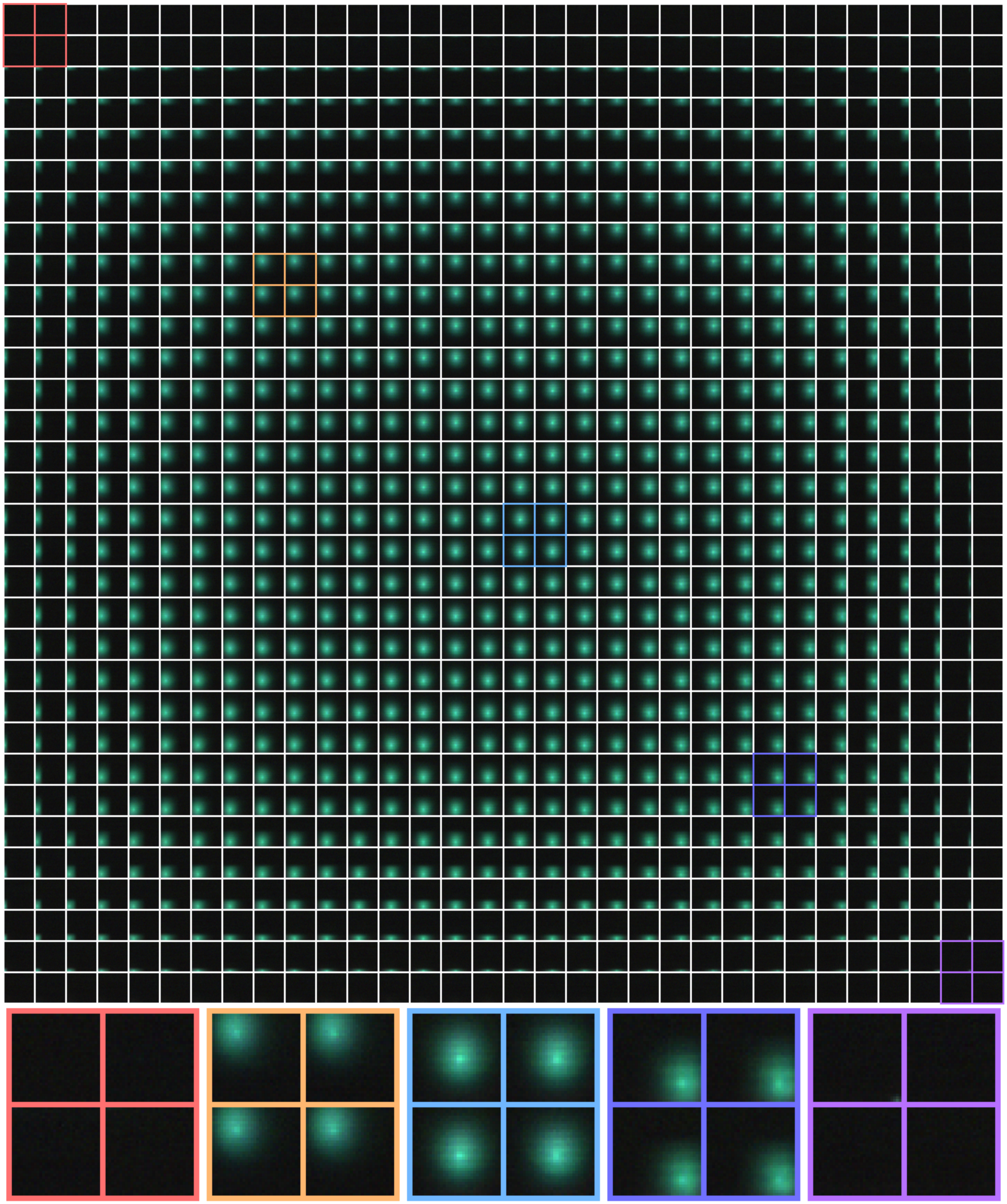

Figure S6. Captured energy distributions with respect to LC1. To capture this light field slice, we set one neuron parameter as 1 and others' as 0 in sequence for LC1; set all neuron parameters as 1 for the input plane; and set all neuron parameters as 1 for LC2. The captured outputs are linearly normalized between 0 and 1 for visualization.

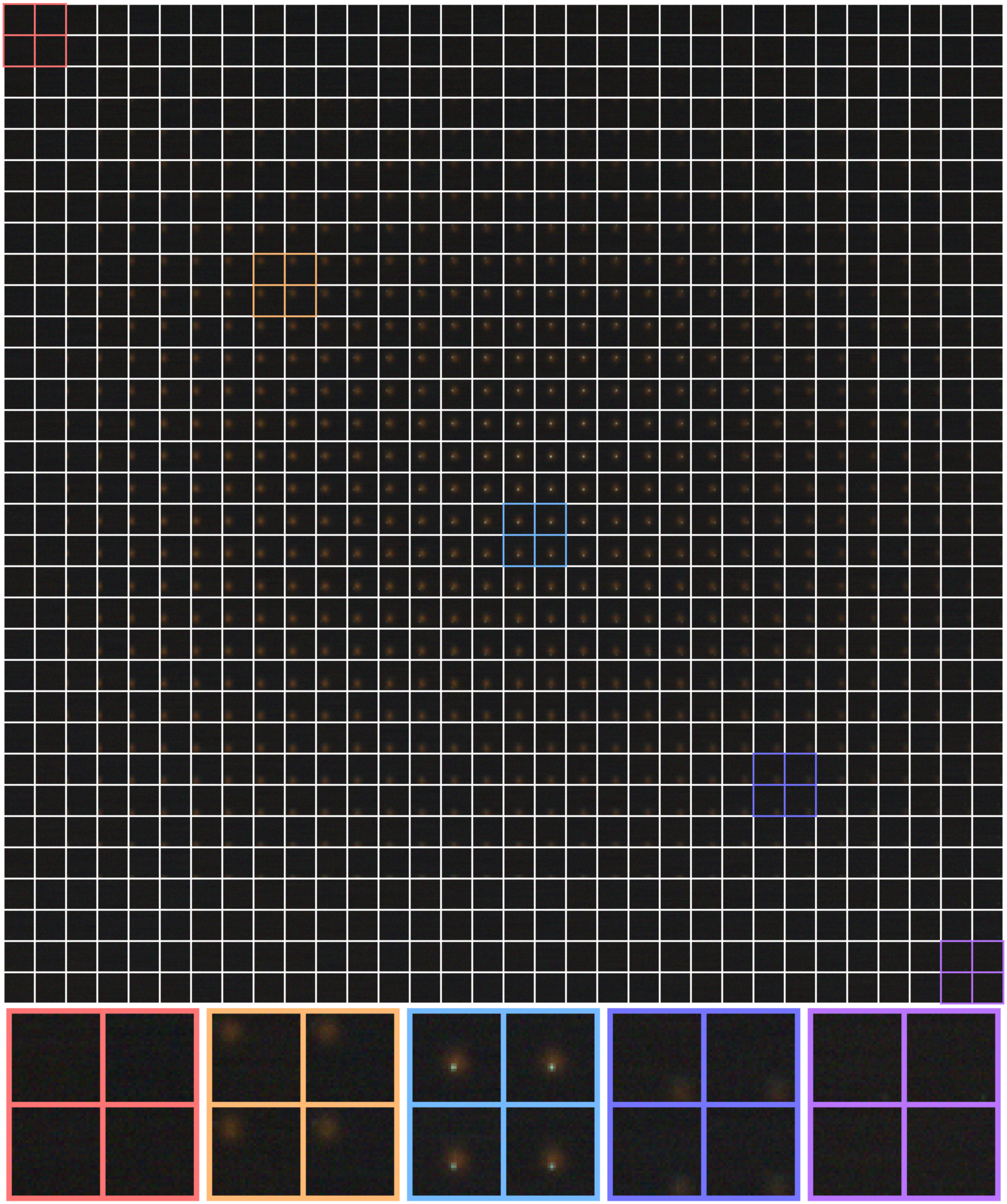

Figure S7. Captured energy distributions of the input plane's center neuron with respect to LC2. To capture this light field slice, we set one neuron parameter as 1 and others' as 0 in sequence for LC2; set the central neuron's parameter as 1 but others' as 0 for the input plane; and set all neuron parameters as 1 for LC1. The captured outputs are linearly normalized between 0 and 1 for visualization.

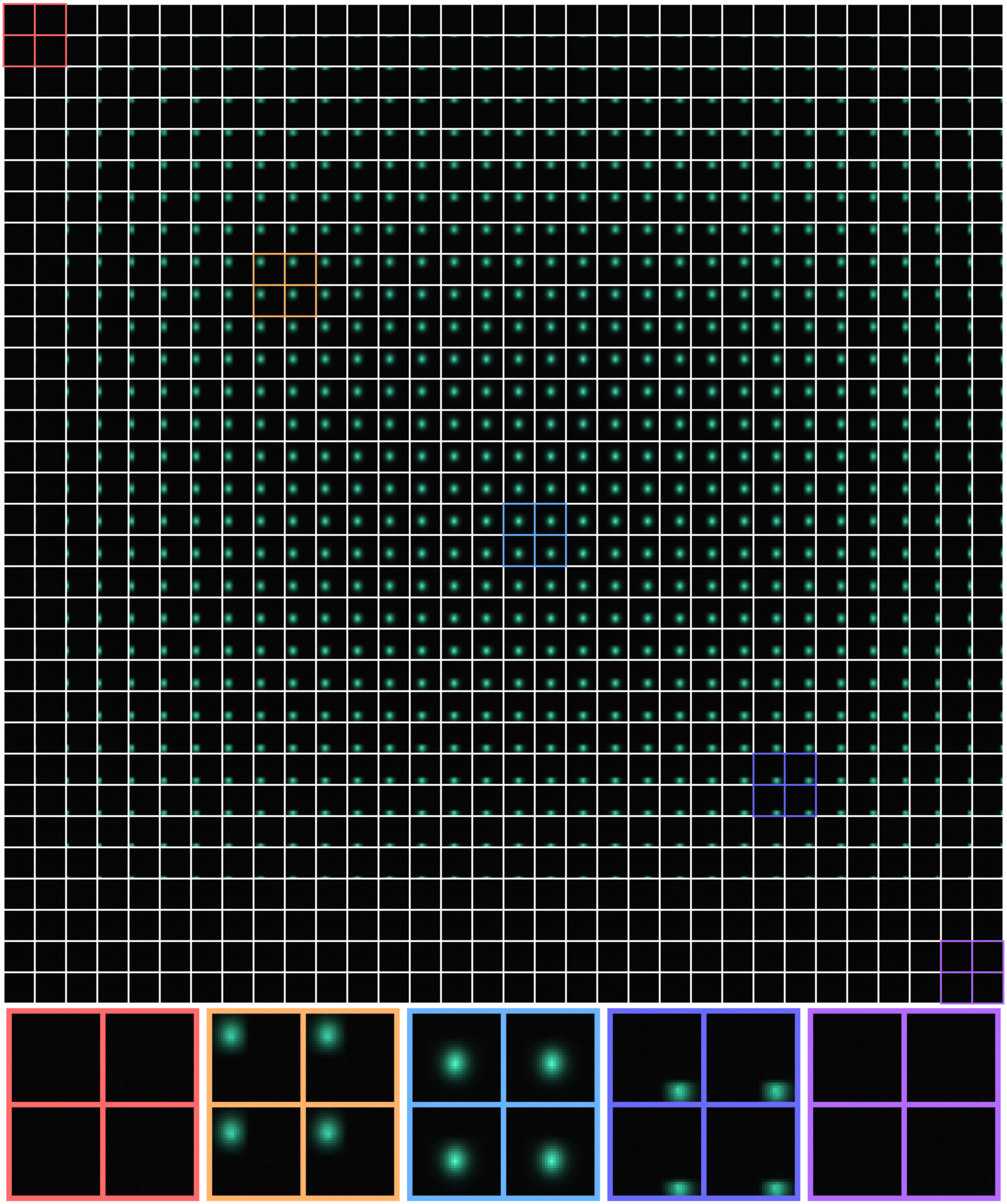

Figure S8. Captured energy distributions with respect to LC2. To capture this light field slice, we set one neuron parameter as 1 and others' as 0 in sequence for LC2; set all neuron parameters as 1 for the input plane; and set all neuron parameters as 1 for LC1. The captured outputs are linearly normalized between 0 and 1 for visualization.

S2.3 Contrast Characterization

The contrasts of the input plane, LC1, and LC2 are measured and visualized in Figure S9, S10, and S11. We apply 256 pixel values varying from 0.0 to 1.0 to each plane's central neuron and capture the corresponding output. As shown in the figures, even though the input parameters range from 0 to 1 with 256 gray levels, the actual valid gray level is less than 256 since the noise is too large to identify similar values. The per-pixel valid gray level is also automatically learned by the FL paradigm, and we do not need to define it explicitly.

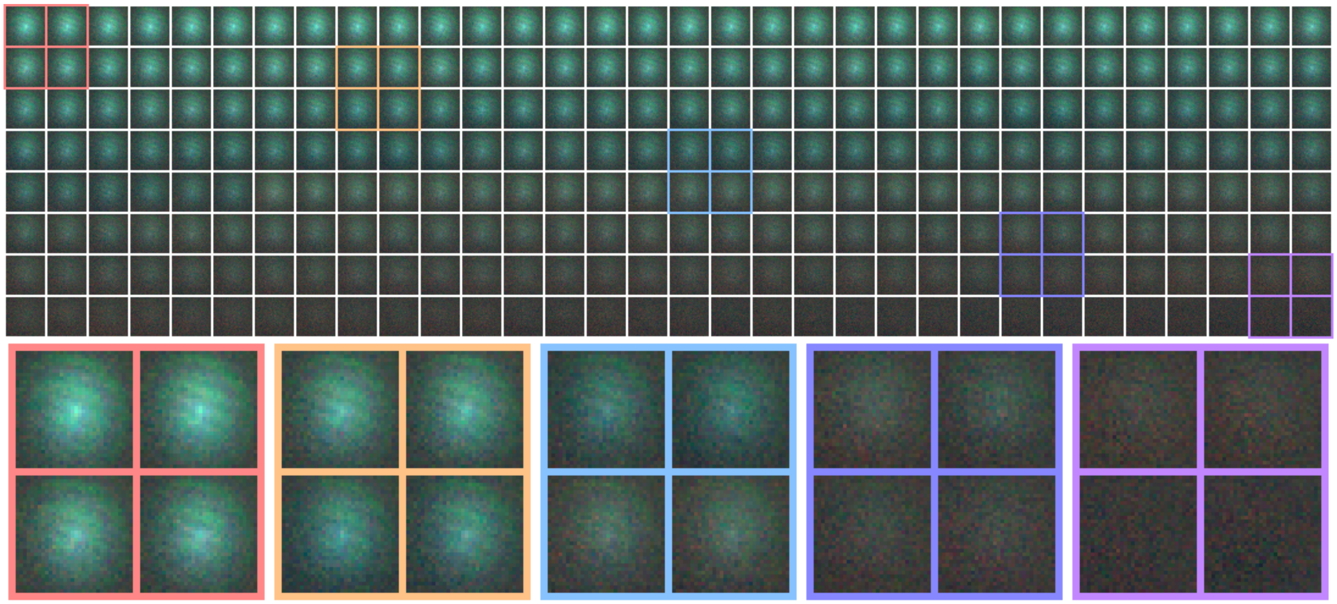

Figure S9. Captured energy distributions of the input plane's central neuron with changing parameter values. To capture this light field slice, we set the central neuron's parameters from 1.0 to 0.0 and others' as 0 in sequence for the input plane; we set all neuron parameters as 1 for LC1 and LC2. The captured outputs are linearly normalized between 0 and 1 for visualization.

S2.4 Linearity Assessment

Liquid crystal panels are considered as linear components disentangled between layers[1] However, we find that the LFNN prototype is not a perfect end-to-end linear system in practice, making it non-differentiable and hard to train. As shown in Figure S12, we fix the parameters of two planes and assess the linearity of the last plane by linearly compositing random parameters. The parameter of the third row is the sum of the first two rows. With a perfect linear system with respect to the tested plane, the captured output should form a linear addition function. The results show significant bias to a linear function. In addition, the bias between the left and right columns varies, implying that the bias is a function of the parameters of the other two planes. In practice, the whole device is a high-dimensional system whose layers are entangled. It is difficult to accurately represent the system by classic explicit or static implicit models. As such, our functional learning paradigm is necessary and works well even in this nonlinear system.

There can be many possible sources of nonlinearity. One known source is the imperfectness of the electronic control circuit and photoelectric conversion of the system. The actual physical response is not always linear to the control parameter. To avoid such a nonlinearity, we composite binary parameters, e.g., each neuron is either 0 or 1, but still observe structured bias, which implies there are other sources of nonlinearity in the system. Other sources could be chromatic dispersion, imperfect manufacture of polarizers, mixed materials with unknown optical properties, or other non-optical sources.

In general, this experiment verifies that there are always gaps between real-world systems and theoretical models. Because the bias sources of the LFNN device are unclear, it is impossible to accurately train such a system in classic ways via numerical models.

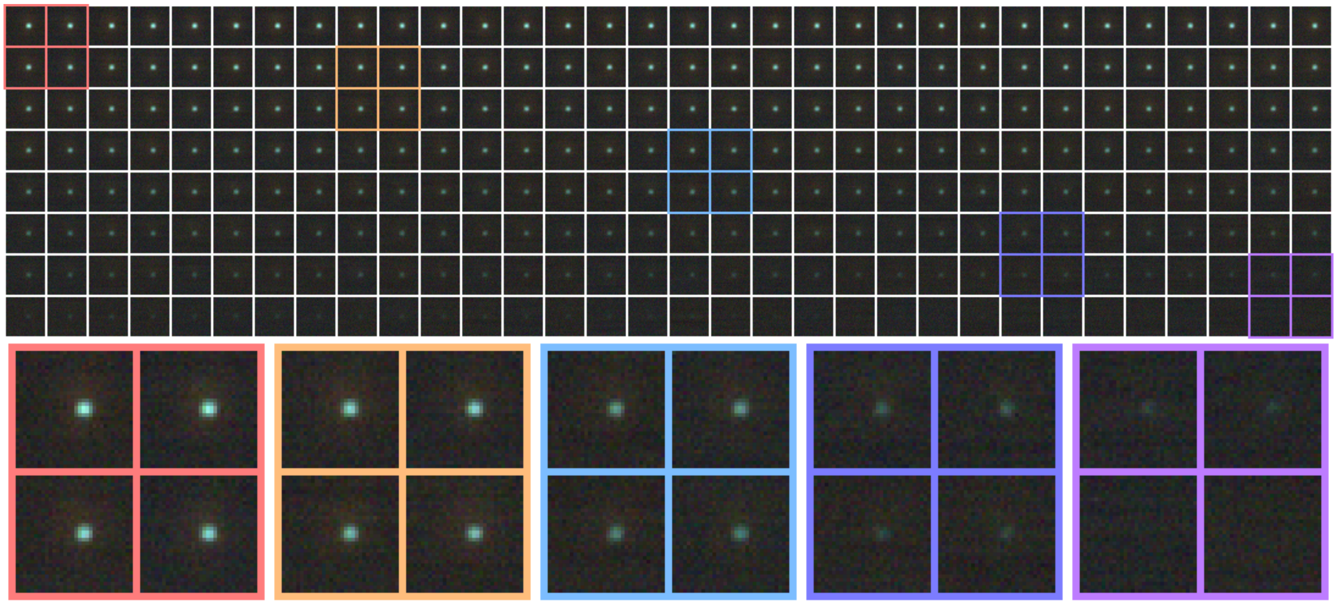

Figure S10. Captured energy distribution of LC1's central neuron with changing parameter values. To capture this light field slice, we set the central neuron's parameters from 1.0 to 0.0 and others' as 0 in sequence for LC1; we set all neuron parameters as 1 for LC2 and set the central neuron's parameter as 1 but others' as 0 for the input plane. The captured outputs are linearly normalized between 0 and 1 for visualization.

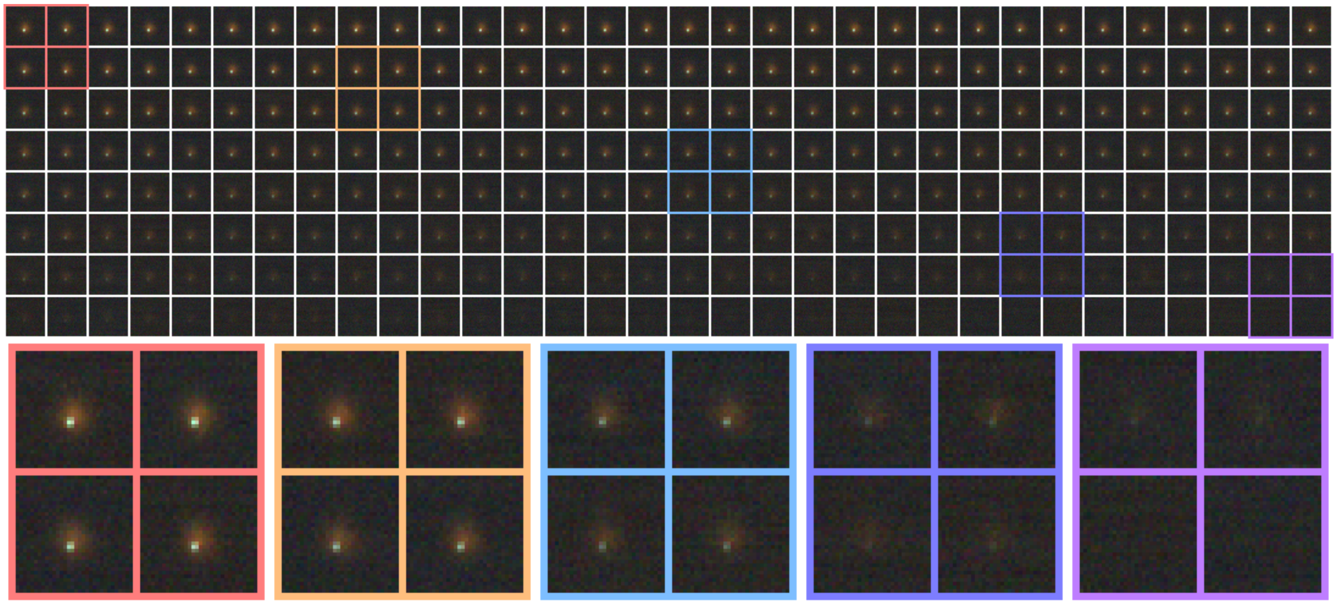

Figure S11. Captured energy distributions of LC2's central neuron with changing parameter values. To capture this light field slice, we set the central neuron's parameters from 1.0 to 0.0 and others' as 0 in sequence for LC2; we set all neuron parameters as 1 for LC1 and set the central neuron's parameter as 1 but others' as 0 for the input plane. The captured outputs are linearly normalized between 0 and 1 for visualization.

S2.5 System Computing Performance

The prototype system is with off-the-shelf components for proof of concept rather than seeking cutting-edge bandwidth. Thus, the components' optical and electrical parameters, such as transmittance, power efficiency, resolution, and response time, are far from the maximum capability of the architecture. According to the resolutions and the frame rates, the computing capability, measuring the total number of operations per second for the architectures, is 796.3

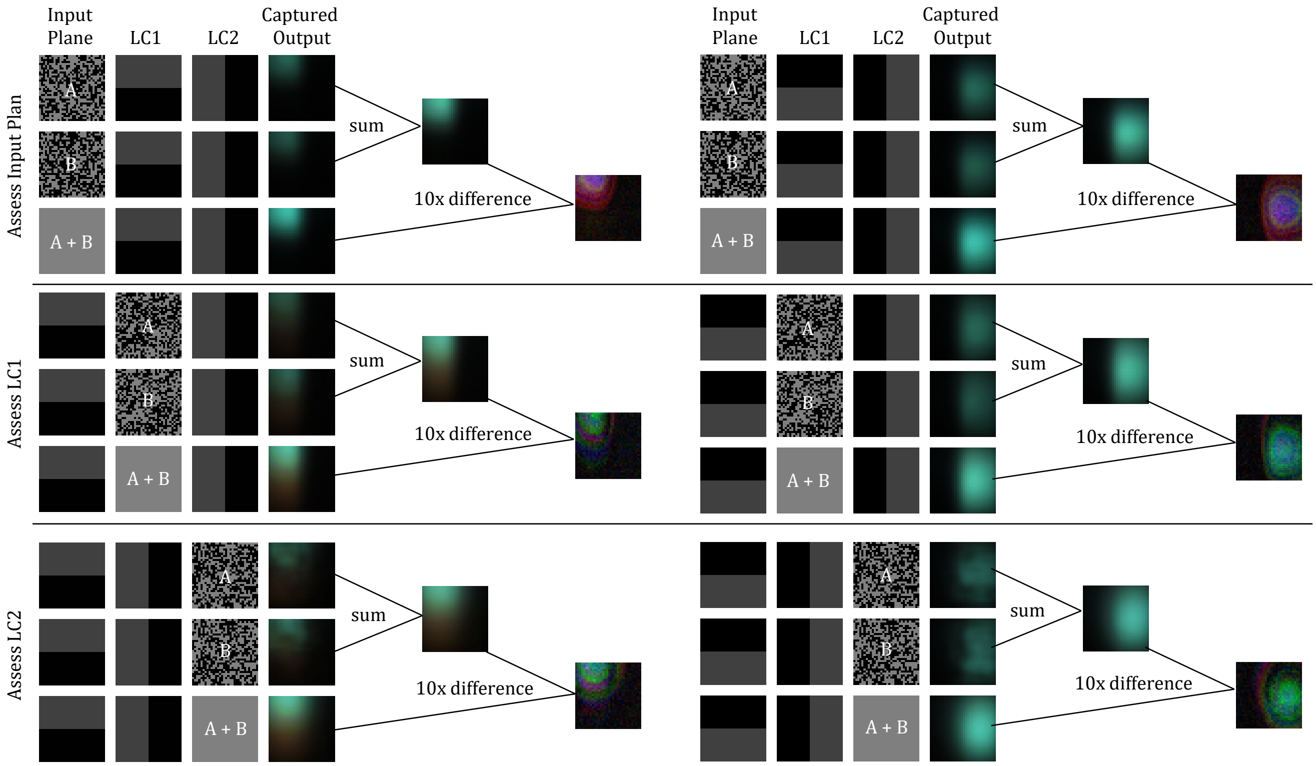

Figure S12. Linearity assessment of the LFNN device. We generate and composite random parameters for the input plane, LC1, and LC2. We change only one plane's parameters to capture three outputs in each test case. For the tested plane, the third row's parameters are equal to the sum of the other two rows' parameters. In theory, the output of the third row should be equal to the sum of the first and second row if the device is a perfect linear system. The captured outputs are linearly normalized between 0 and 1 for visualization.

tera-operations per second (TOPS). However, using the current training paradigm to train such a huge neural network system is impractical in terms of time. Therefore, we only use a small portion of the computing capability. Specifically, we merge multiple LC neurons as one by sharing the weights to reduce the training parameters. The reduced model enables 1.13 giga-operations per second (GOPS). The power consumption of all the optical devices, including the light source, LCD panels, and the camera, is 37.275 watts. In addition, we use a computer to control the system, whose power consumption is 58 watts. In general, the primary challenge to fully utilizing the computing capability, i.e., not only simply using it, but also processing complex high-resolution inputs, is developing new training algorithms, such as integrating CNNs, to reduce the training parameters. For improving computing capability and reducing power consumption, potential directions include using high-transmittance optical components, specified low-power control IC, high-speed camera, and light source based on laser or micro-LED.

S3 Evaluation

S3.1 Functional Learning

To evaluate functional learning, we test different training paradigms on our LFNN prototype for the 1-layer MNIST classification task.

The first test is to verify the update of Z-data. While the iterative *z-* and *p-learning* strategy shows clear effect on the convergence and accuracy (Section S4.1), it is unclear to see the effect of update of *z-data* at each epoch. Therefore, we test stopping the update of *z-data* after 300 epoch and continue to finish the following epochs. The accuracy of the FNN predicted result is 92.93%, but the LFNN captured accuracy is 38.28%. The experiment indicates that the *z-data* update is necessary for the convergence between the FNN and the LFNN.

Second, we test the different numbers of captured samples for the update of *z-data* per epoch. The accuracy of capturing 128, 256, 512, and 1024 samples is 90.43%, 90.50%, 90.82%, and 91.02%, respectively. Because the improvement gradually decreases, but time consumption exponentially grows, we choose 1024 to train our LFNN prototype to balance performance and speed. The choice of this hyper-parameter depends on the actual device and time budget.

The third test is to verify the decoupling of *p-variable* and *z-variable* in *z-learning* and *p-learning*. We simultaneously update both *p-* and *z-variable* in each sub-problem, and the accuracy is only 7.187%. Clearly, the problem is unsolvable if we do not decouple *p-variable* and *z-variable*.

Finally, we try using different training paradigms to train 1-layer LFNN for MNIST classification task and compare the results. The compared approaches include forward model, genetic algorithm, finite difference, and our functional learning. These paradigms represent optimizing the hardware with explicit (but not accurate) gradient, without gradient, with measured gradient, and with the implicit gradient.

S3.2 X-activation

The validity of X-activation is evaluated with the 3-layer CIFAR10 classification task. With X-activation, the FNN predicted accuracy is 44.97% and the LFNN captured accuracy is 43.66% at 1800 epoch. Without X-activation, the accuracy drops to 39.19% and 38.82% for FNN and LFNN, respectively. The lack of negative operators does restrain the performance of incoherent ONN, and the X-activation evidently enhances the inference capability. However, using only trivial nonlinear activation is also an option that balances the performance and the cost, which also can be robustly trained by the FL as shown in Figure S13.

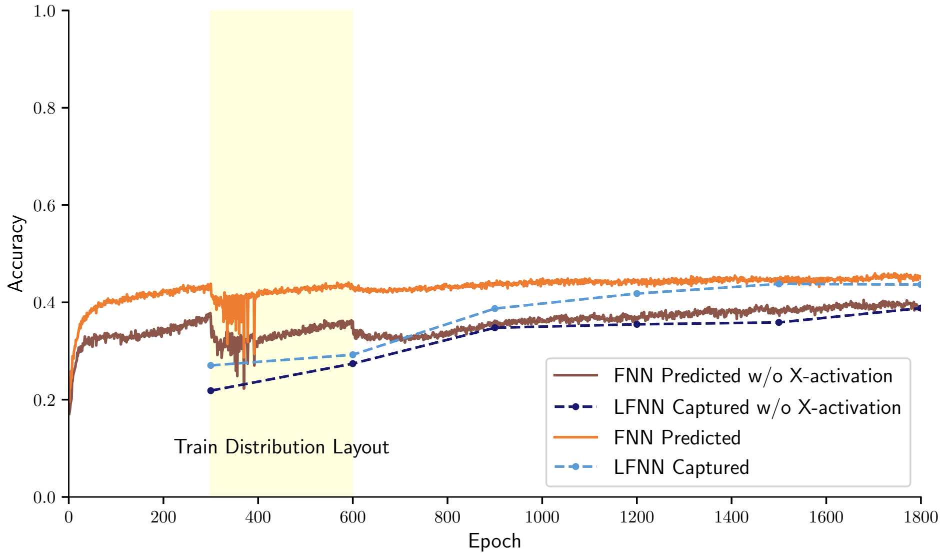

Figure S13. Log of training the 3-layer LFNN for CIFAR10 classification task with or without X-activation. The training of distribution layout is discussed in Section S4.

S3.3 Comparison of Training Paradigms

We compare the functional learning paradigm with existing machine learning paradigms to verify its efficacy Table S2. The test is conducted using our LFNN prototype for the 1-layer MNIST classification task. The chosen paradigms

Table S2. Classification accuracy of training 1-layer LFNN for MNIST clalssification task using different training paradigms. The tested device is 1-layer LFNN consisting of 2 LC panels. The results of the genetic algorithm, the finite difference, and the functional learning paradigms are measured using approximately equal training time.

	Forward Model	Genetic Algorithm	Finite Difference	Functional Learning (Ours)
**Gradient**	explicit	none	measured	implicit
**1-Layer MNIST**	23.50%	14.06%	8.594%	90.78%

include functional learning, forward model, finite difference, and genetic algorithm. Note that the setting and result of this experiment are different from that of the complete training we report in the main manuscript and Section S4. Here we only train the epochs before training distribution layout, i.e., 100 epochs for 1-layer LFNN. The reason is that using the pruning scheme (Section S4) to train the distribution layout requires the network weights to converge to a relatively stable status that we can prune unimportant connections. However, the finite difference and generic algorithm cannot converge to a stable status within a practical training time. In order to make the comparison fair, we do not use pruning to all paradigms in this experiment. For the genetic algorithm, finite difference, and the functional learning paradigms, capturing LFNN outputs dominates the training time. Therefore we measure these three paradigms using 1024×100 captured outputs which can train 100 epochs for the functional learning paradigm. The overall results are reported in Table S2.

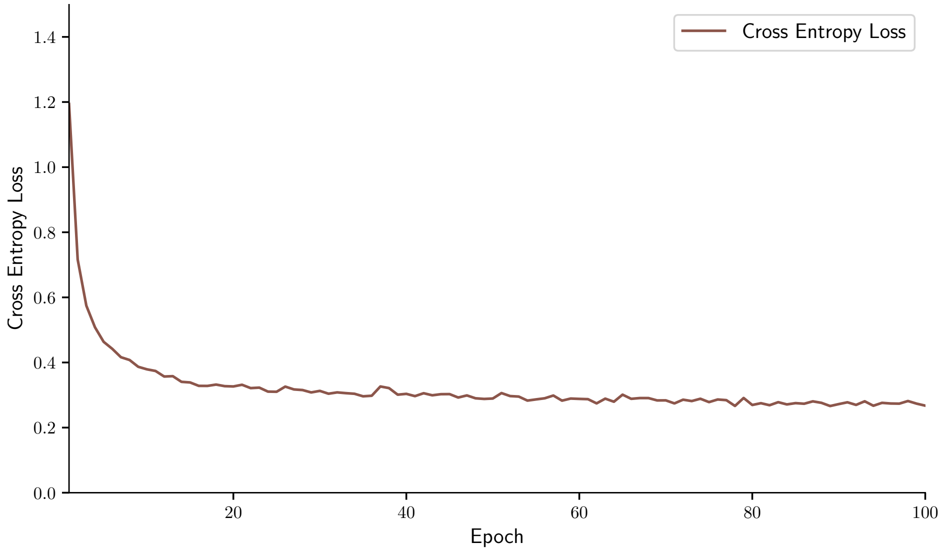

Figure S14. Loss of training 1-layer LFNN for MNIST classification task using functional learning.

Using analytical forward model[1] with the back-propagation algorithm is an intuitive choice to train the parameters of the LCs. This paradigm is commonly used in training ONNs[2]. The training process is similar to removing the *z-learning* part. However, manufacturing and installment unavoidably introduce system bias. Treating the misalignment as a random variable of the training paradigm can alleviate the misalignment[3]. Similarly, we measure the actual physics, e.g., noise, misalignment, and narrow viewing angles, of the LFNN to build a forward model for the training. Specifically, we first measure the point spreading function (PSF) of every input neuron with respect to every output neuron. Then we measure and model the impact of every LC neuron on every input neuron's PSF as a linear function by switching on and off of the corresponding neurons and recording the change on the output plane. In order to calibrate the linearity of control parameters, we imitate gamma calibration and build a lookup table for each panel by measuring the actual system output. Even though we build a forward model to approximate the LFNN system, the training yields a low prediction accuracy since the actual physics contains thousands of correlated parameters (Section S2.4), forming a high-dimensional state space that cannot be measured in practice. While the train losses of these two paradigms are generally comparable, as shown in Figure S14 and Figure S15, there is a clear gap between the actual prediction accuracy. Even though we use the measured forward model, it is still not enough to train a complicated system like the LFNN.

While the finite difference technology fits systems with a small number of parameters[4], we found its performance

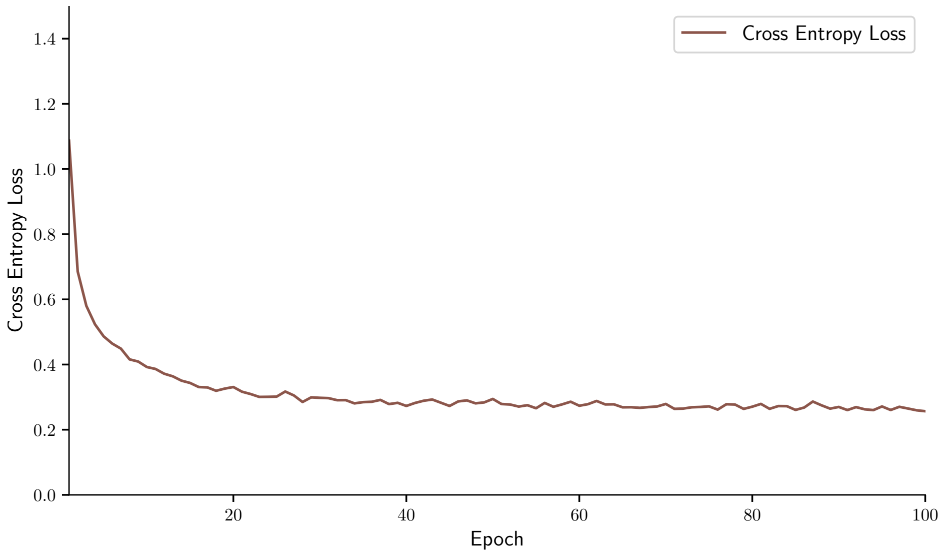

Figure S15. Loss of training 1-layer LFNN for MNIST classification task using forward model.

dramatically drops if the number of parameters increases. The finite difference paradigm relies on measuring the actual finite difference as gradients to optimize the parameters. While facing thousands of parameters, it has to measure the finite difference of every parameter, which requires capturing 6144 output images from the LFNN to calculate the gradient and update the parameters at each epoch. Another difficulty is that the gradient is related to the input image. In order to make the experiment realizable, we randomly draw one input image to calculate the gradient at each epoch. In contrast, our FNN paradigm captures 1024 output images to implicitly evaluate the gradients for 60000 images at each epoch. As a result, using the finite difference paradigm yields a 8.594% prediction accuracy within equal training time. Here we test all learning rates of $0.0001, 0.0005, 0.001, 0.005, 0.01, 0.05, 0.1, 0.5$ and report the best accuracy using 0.001. We plot the train loss in Figure S16. As expected, there are vibrates because the measured gradient is calculated from the noisy output of one input image. More importantly, measuring gradient for a large number of parameters is too time-consuming and thus only 16 epochs can be conducted within equal training time.

Genetic algorithm is a stochastic paradigm that can be used in training neural network parameters[5]. While converging efficiently with a small number of parameters, stochastically exploring a high-dimensional space without

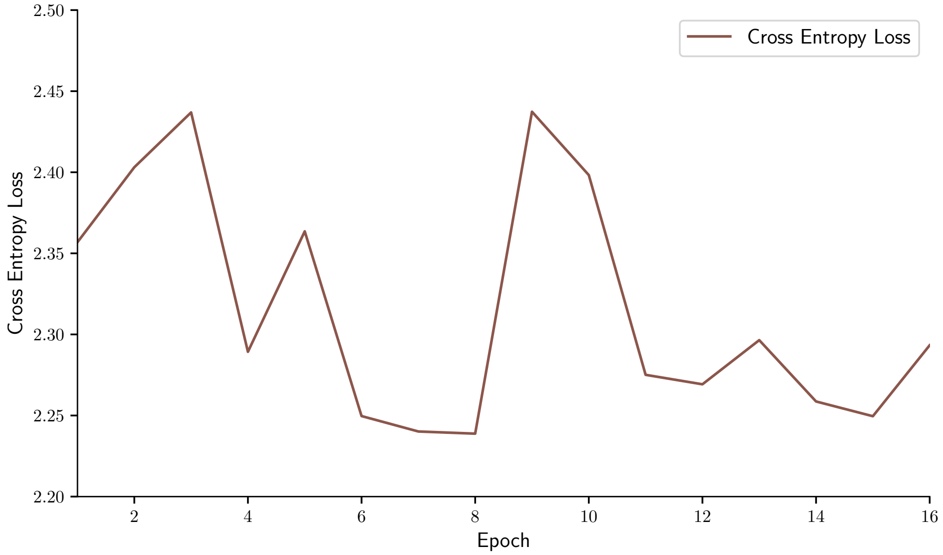

Figure S16. Loss of training 1-layer LFNN for MNIST classification task using finite difference.

gradients is futile. We leverage the scikit-opt genetic algorithm train LFNN. The population size is set to 8 and the mutation probability is set to 0.001. The metric of evaluating an offspring is calculated by randomly drawing 128 images and capturing the outputs. Under this setup, the genetic algorithm requires 1024 images at each epoch, which is the same as our FL configuration. Within equal training time (100 epochs), the prediction accuracy is 14.06%. As shown in Figure S17, the loss decreases steadier compared with that of the finite difference paradigm. However, exploring a high-dimensional solution space without gradients is inefficient because there are too many directions, resulting in a low speed of convergence. With the guidance of gradient, the other tree paradigm's losses drop to small numbers in the first epoch in comparison.

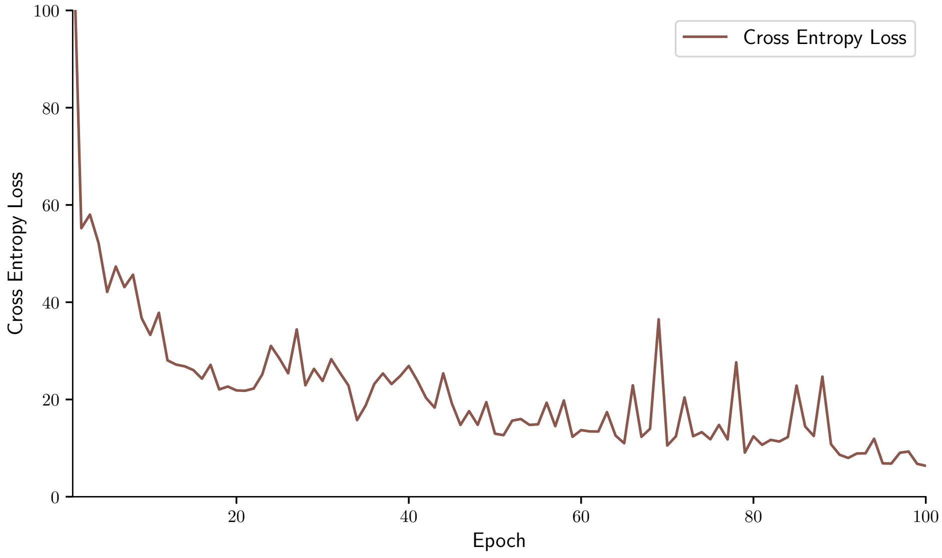

Figure S17. Loss of training 1-layer LFNN for MNIST classification task using genetic algorithm.

S3.4 Functional Neural Network Output

In order to evaluate the capability of the FNN in terms of reflecting the real physics, we compare the predicted output of the FNN and the actual output of the LFNN. Figure S18 shows the FNN's predicted point spreading functions of input neurons, which is supposed to match the results of Figure S4. Figure S19 is the difference between the predicted and captured outputs. As can be seen, the FNN can well approximate the real LFNN output except for a few noises.

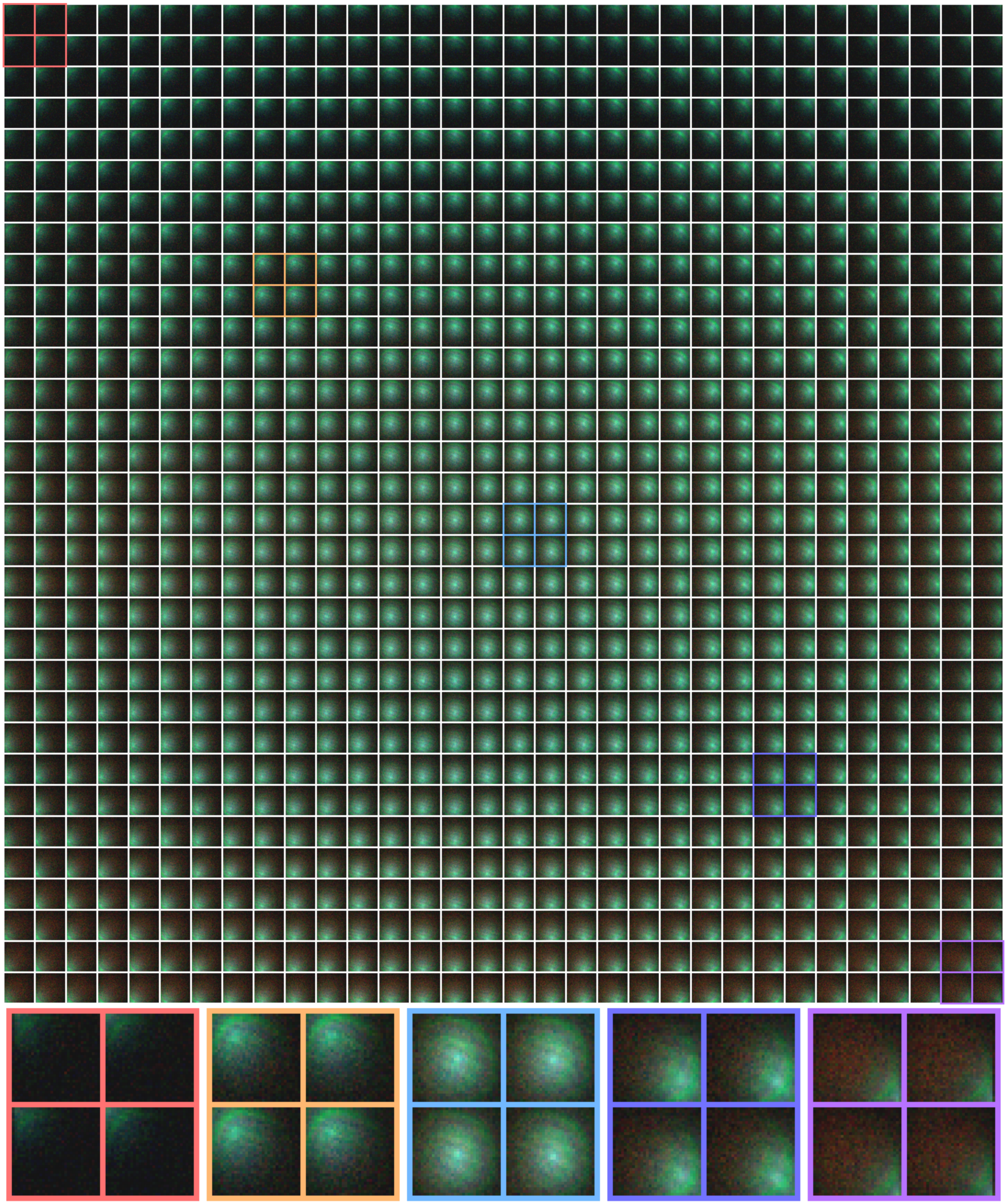

Figure S18. The FNN's predicted energy distributions of input plane's neurons. To capture this light field slice, we set one neuron parameter as 1 and others' as 0 in sequence for the input plane; set all neuron parameters as 1 for LC1; and set all neuron parameters as 1 for LC2. The captured outputs are linearly normalized between 0 and 1 for visualization.

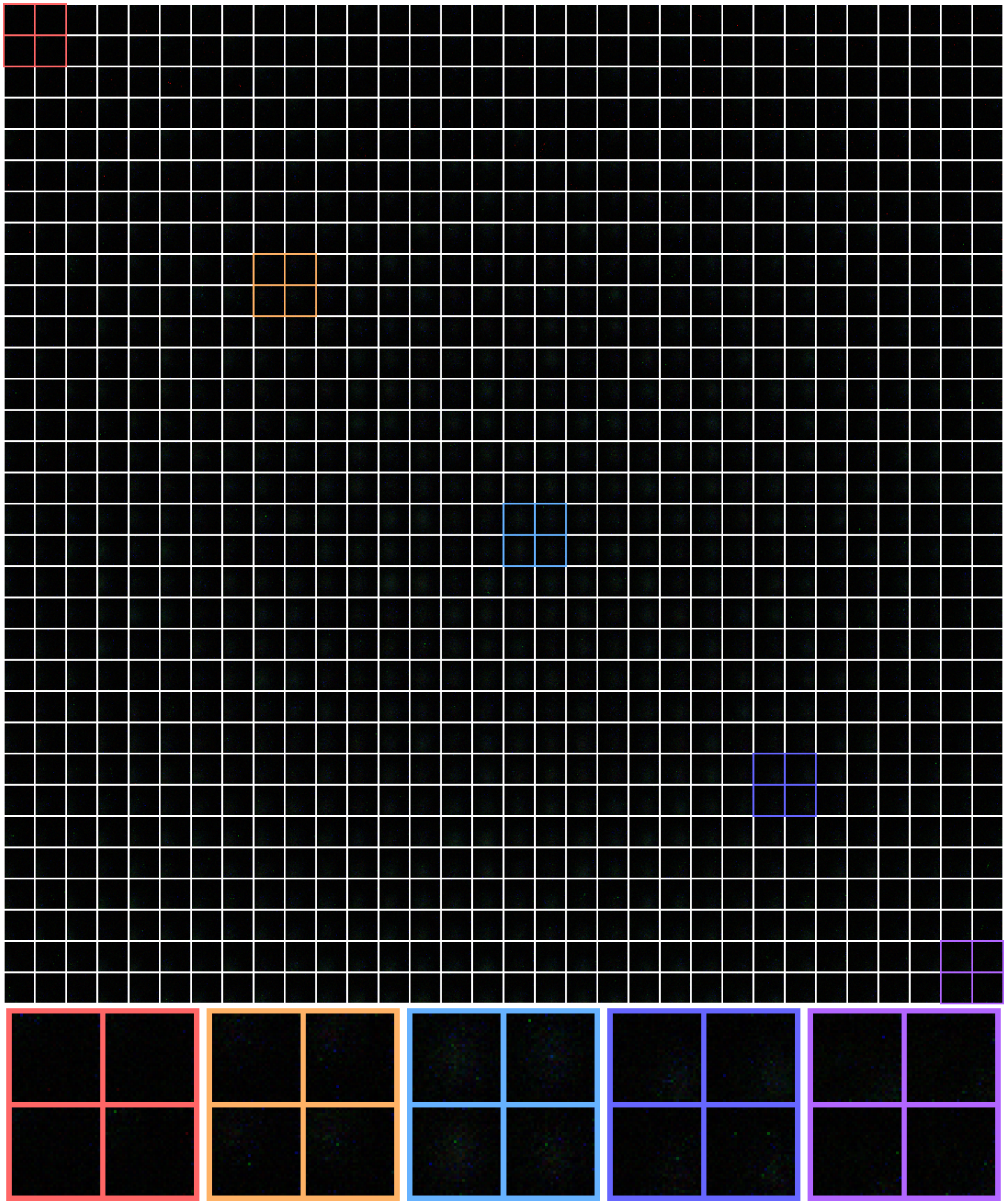

Figure S19. The difference between the FNN's predicted output (Figure S18) and the LFNN's captured output (Figure S4). The average relative MSE is 0.000413.

S4 Experiments

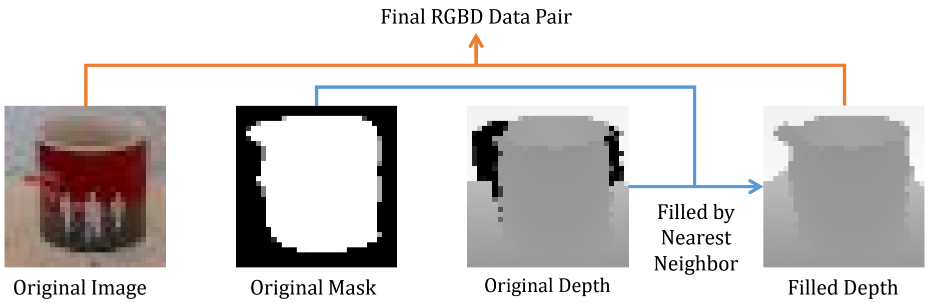

Figure S20. Preprocessing of the RGBD data.

We test objects classification, object detections, and single-image depth estimation on our LFNN prototype. The training is conducted using the proposed FL paradigm on a computer with one Intel i7 9700K CPU, one RTX 2080 Ti GPU, and 32 GB physical memory. The deep learning framework is PyTorch.

For the classification applications, we use the MNIST dataset[6] to test the 1- and 2-layer LFNN, and the CIFAR10 dataset[7] to test the 3-layer LFNN. The MNIST dataset has 60,000 examples in the training set and 10,000 examples in the test set. The CIFAR10 dataset has 50,000 images in the training set and 10,000 images in the test set. The classification accuracy is summarized in Table S6.

Digit recognition and object recognition tasks are conducted in MNIST and CIFAR10 data set using the 1-layer LFNN. To train the LFNN device to recognize a target, we use all positive samples in the data set and sample the same number of negative samples. For example, we use 6,000 digit 0 samples and randomly pick 6,000 digit 1 to 9 samples for the training of digit 0 recognition. The test set is sampled by the same strategy to make positive and negative sample sizes match.

The 'coffee mug' category of an RGB-D dataset[8] is used to test the 4-layer LFNN for the depth estimation task. The original depth data is scanned by the Kinect and has many holes. We use the original mask labels of the dataset to separate the object and background layers, then use a nearest-neighbor-searching scheme to fill the holes in each layer (Figure S20). However, the original mask labels are not very accurate and thus introduce some errors to the object shapes. There are total 4,500 such RGBD pairs in the training set and 300 pairs in the test set.

We use the cross-entropy loss term for the *p-learning* of classification and recognition tasks, and the L1 loss for the depth estimation task. The optimization algorithm is Adam[9], and the learning rate is 0.001. Because we use a commercial low-speed camera (four frames per second) to implement the LFNN prototype, the performance bottleneck of the training process is the time consumed by capturing *z-data*. Therefore, the average training time of one epoch is about 4 minutes. The total training time for each task can be easily calculated given their total epochs reported in this section.

For the classification tasks, the predicted probabilities are represented by different distribution layouts. Specifically, a class probability is the intensity sum of relevant areas on the output plane. One naive probability distribution layout is to divide the plane into a regular grid and use one cell to represent the probability of one class. For example, using the intensity of ten light spots to represent the classification probability of ten hand-written digits on the MNIST data set[2]. Because manually optimizing the probability distribution layout requires an explicit model of the LFNN, there is no trivial algorithm to do so. We decide to make the probability layout distribution also trainable. We find that splitting a large spot into four small spots enables large optimization space for the

Table S3. Classification accuracy of the LFNN captured output, the FNN predicted LFNN output, and the equal-layer digital dense neural network (DNN) output. The LFNN has 12,288 trainable variables per layer. The FNN has 28,438,144 trainable variables per layer. The digital dense neural network comprises dense layers connected by ReLU with neuron sizes of (784,10), (784,784,10), and (3072,3072,3072,10), respectively.

	LFNN Captured	FNN Predicted	Digital DNN
**1-Layer MNIST**	91.02%	91.39%	92.71%
**2-Layer MNIST**	94.77%	95.45%	98.32%
**3-Layer CIFAR10**	45.62%	46.19%	53.62%

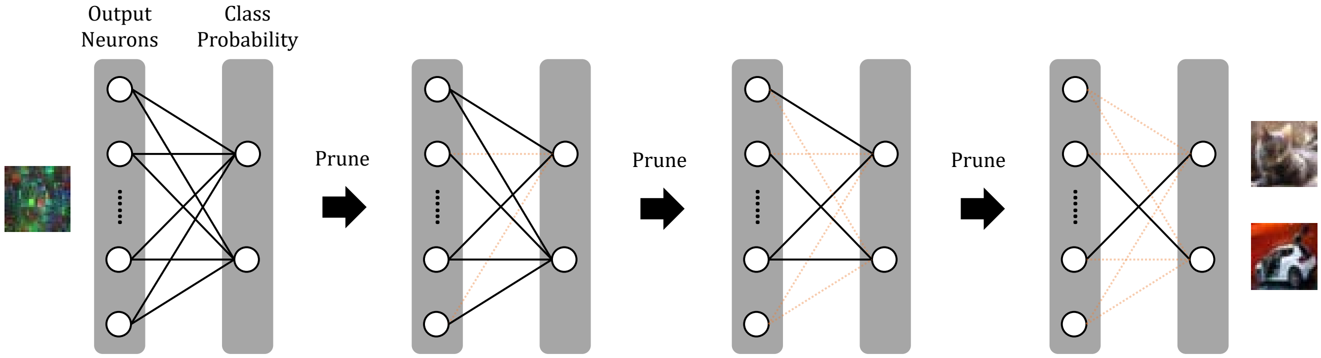

Figure S21. Illustration of the process of training the distribution layout. This figure is an example of training the distribution layout for two classes. To begin with, we divide the output plane into 64 grids to represent output neurons. Every output neuron is connected to every class probability. The connections are trained during the training for classification. At the end of every epoch, each class probability will prune one least weighted connection linked to it. We repeat this process until only a target number of connections left for each class probability. Finally, the weights of the left connections are set to 1, so the sum of intensity in different regions of the output plane represents the predicted probabilities of different classes.

probability distribution layout. For example, the feature on one corner of the input plane might not be able to reach the other side of the output image due to limited scattering angles, but using multiple gathering spots on the output plane can mitigate this problem. Instead of assigning a handcrafted layout, we make the probability distribution layout trainable to adapt the optical property of the LFNN prototype. This modification improves the accuracy of 1-layer MNIST classification from 87.69% to 91.02% Figure S21 illustrates the process of training the probability distribution layout. To begin with, every neuron on the last output plane is digitally connected to all class probabilities. The predicted class probabilities are the sum of output neurons through these connections. These connections are updated during the training process. At the end of every epoch, the least weighted connection to one class probability is pruned. We repeat this process until only a target number of connections left for each class probability. Because our LFNN prototype's input neurons have narrow scattering angles, we leave four dispersed connections to each class. Finally, the left connections' weights are set to 1, so the corresponding areas' intensity sum represents the class's probability. This distribution layout learning scheme makes the classification task adaptive to the actual hardware.

For the depth estimation task, the captured output's RGB channels are added up to the predicted depth.

S4.1 Results of MNIST Classification (1-Layer)

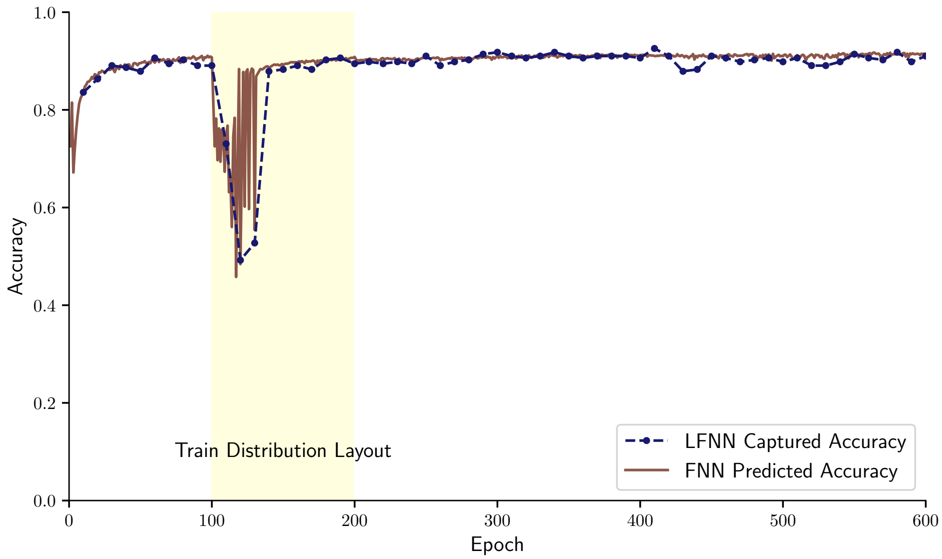

Figure S22. Evaluation of LFNN captured and FNN predicted results for MNIST Classification (1-Layer). While training the distribution layout, some connections are pruned and might lead to a dramatic change in the probability distribution layout, leading to a sudden accuracy decrease. These pruned connections will be replaced by other connections later, and the accuracy gradually recovers after the pruning.

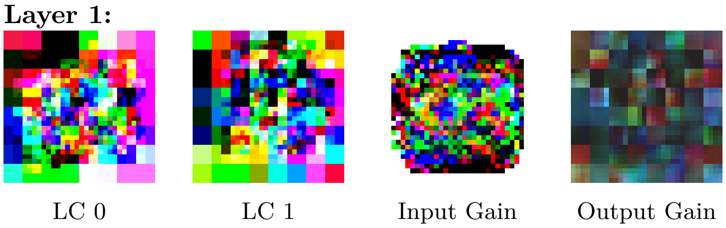

Figure S23. Hardware parameters of MNIST Classification (1 Layer).

Test case 1, the first row is LFNN captured outputs and the second row is FNN predicted outputs.

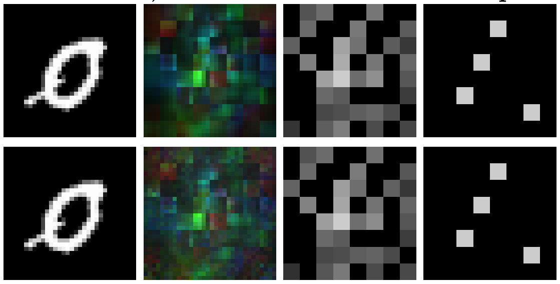

Input 1 Output 1 Distribution Reference

Test case 2, the first row is LFNN captured outputs and the second row is FNN predicted outputs.

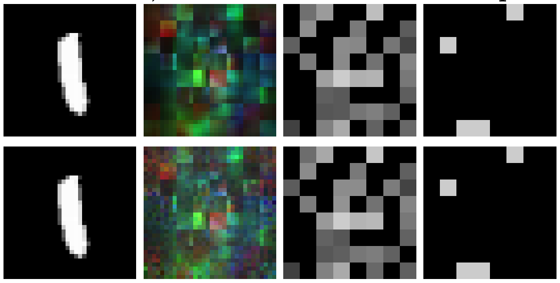

Input 1 Output 1 Distribution Reference

Test case 3, the first row is LFNN captured outputs and the second row is FNN predicted outputs.

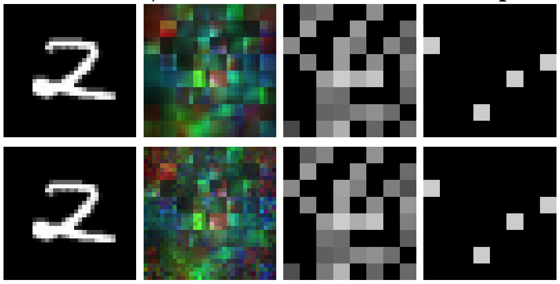

Input 1 Output 1 Distribution Reference

Test case 4, the first row is LFNN captured outputs and the second row is FNN predicted outputs.

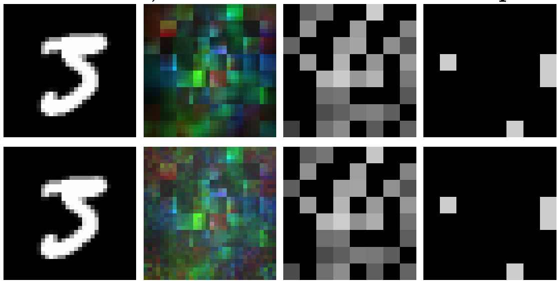

Input 1 Output 1 Distribution Reference

Test case 5, the first row is LFNN captured outputs and the second row is FNN predicted outputs.

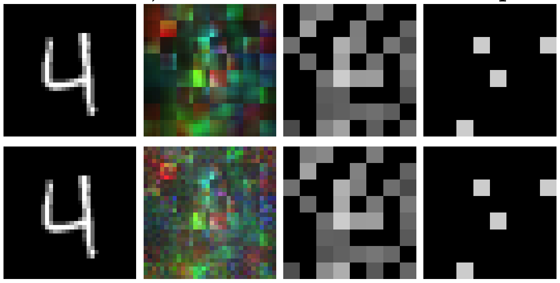

Input 1 Output 1 Distribution Reference

Figure S24. Layer outputs of MNIST Classification (1 Layer) for test cases 1 to 5.

Test case 6, the first row is LFNN captured outputs and the second row is FNN predicted outputs.

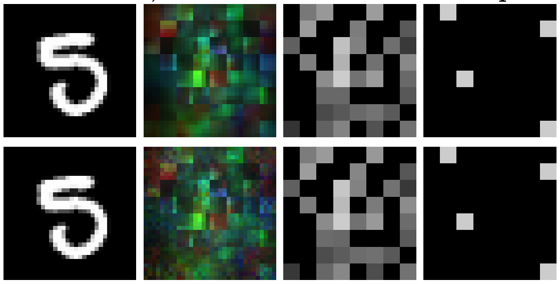

Input 1 Output 1 Distribution Reference

Test case 7, the first row is LFNN captured outputs and the second row is FNN predicted outputs.

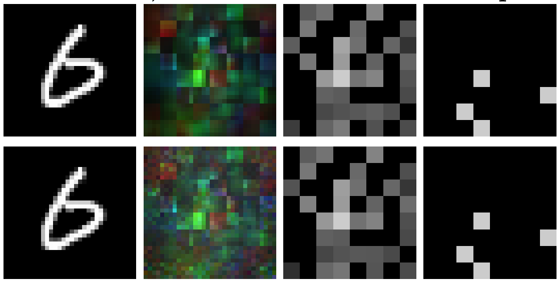

Input 1 Output 1 Distribution Reference

Test case 8, the first row is LFNN captured outputs and the second row is FNN predicted outputs.

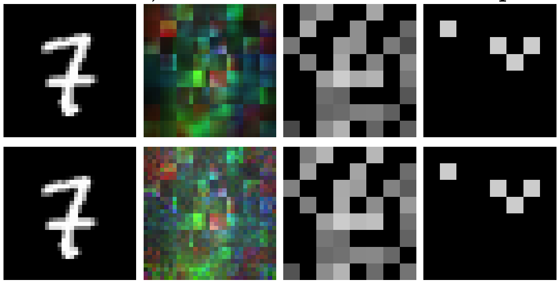

Input 1 Output 1 Distribution Reference

Test case 9, the first row is LFNN captured outputs and the second row is FNN predicted outputs.

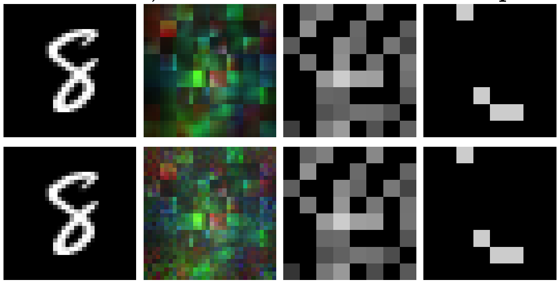

Input 1 Output 1 Distribution Reference

Test case 10, the first row is LFNN captured outputs and the second row is FNN predicted outputs.

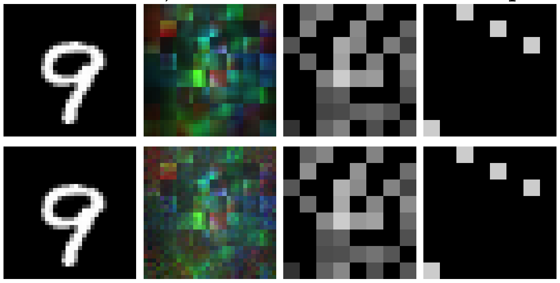

Input 1 Output 1 Distribution Reference

Figure S25. Layer outputs of MNIST Classification (1 Layer) for test cases 6 to 10.

S4.2 Results of MNIST Classification (2 Layer)

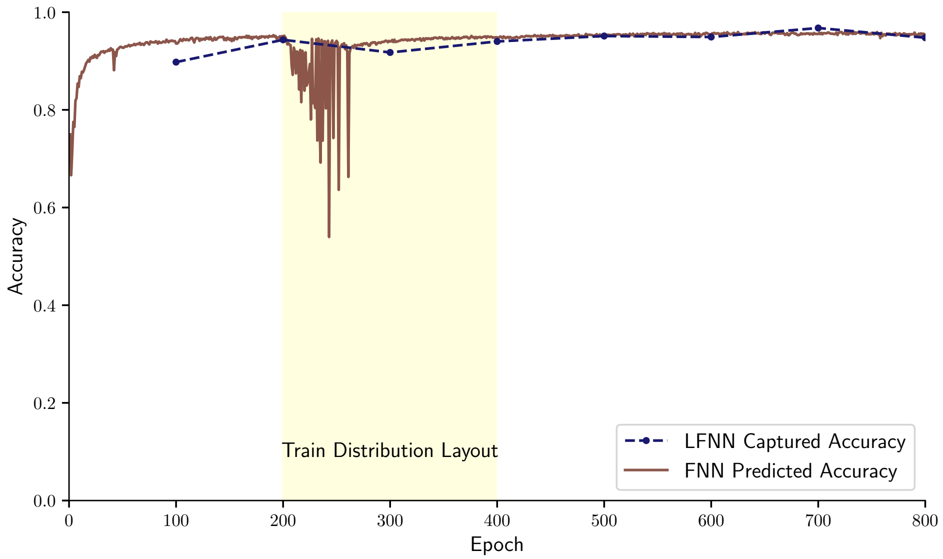

Figure S26. Evaluation of LFNN captured and FNN predicted results for MNIST Classification (2 Layer).

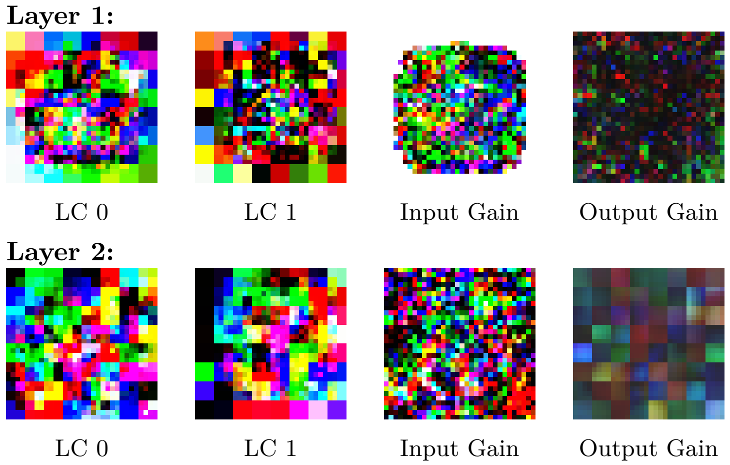

Figure S27. Hardware parameters of MNIST Classification (2 Layer).

Test case 1, the first row is LFNN captured outputs and the second row is FNN predicted outputs.

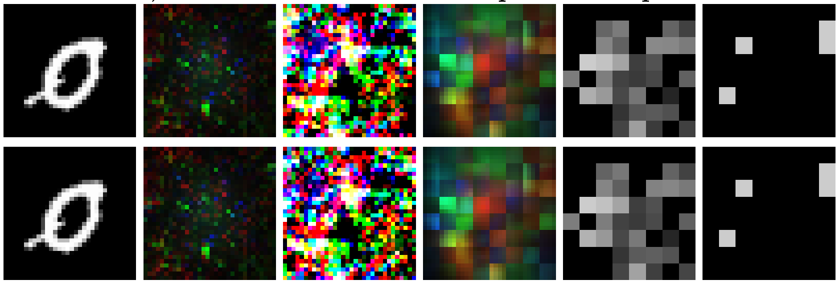

Input 1 Output 1 Input 2 Output 2 Distribution Reference

Test case 2, the first row is LFNN captured outputs and the second row is FNN predicted outputs.

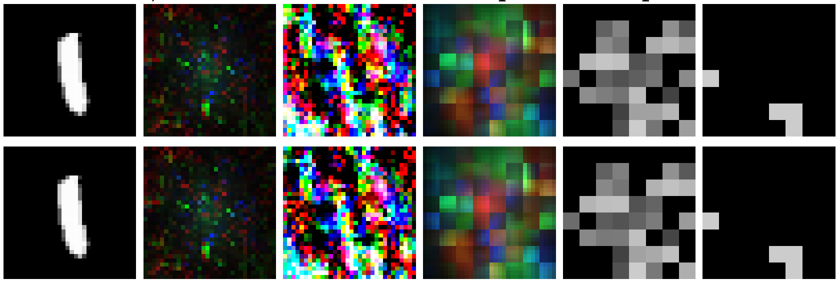

Input 1 Output 1 Input 2 Output 2 Distribution Reference

Test case 3, the first row is LFNN captured outputs and the second row is FNN predicted outputs.

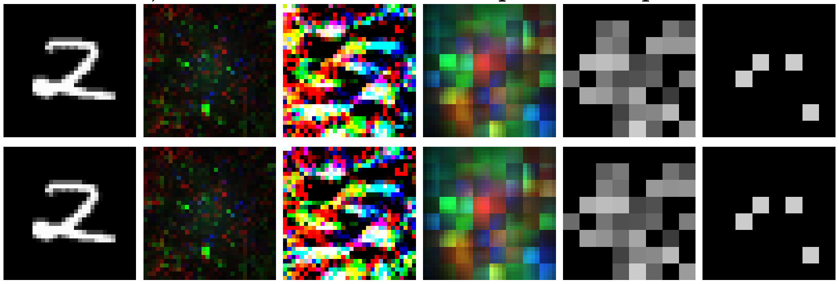

Input 1 Output 1 Input 2 Output 2 Distribution Reference

Test case 4, the first row is LFNN captured outputs and the second row is FNN predicted outputs.

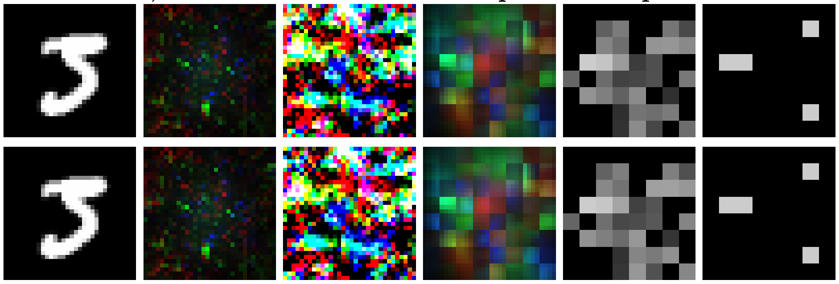

Input 1 Output 1 Input 2 Output 2 Distribution Reference

Test case 5, the first row is LFNN captured outputs and the second row is FNN predicted outputs.

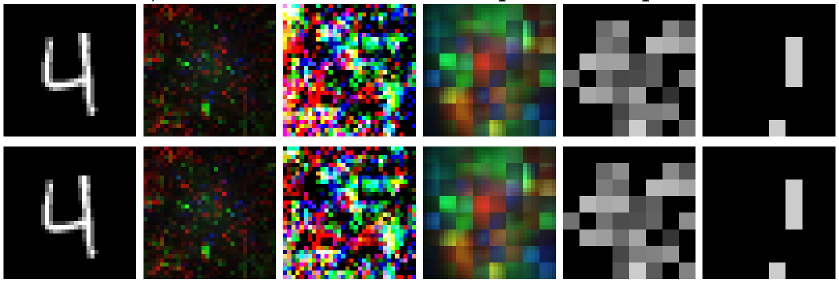

Input 1 Output 1 Input 2 Output 2 Distribution Reference

Figure S28. Layer outputs of MNIST Classification (2 Layer) for test cases 1 to 5.

Test case 6, the first row is LFNN captured outputs and the second row is FNN predicted outputs.

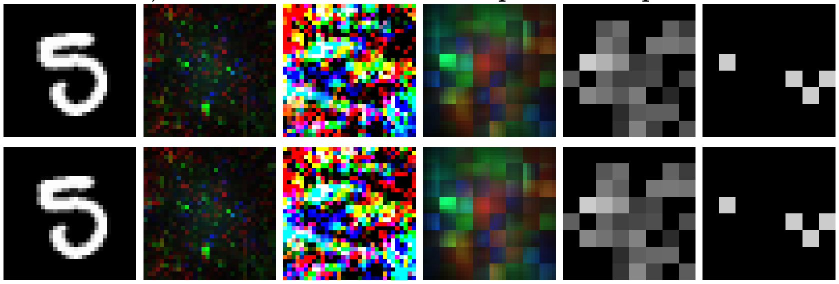

Input 1 Output 1 Input 2 Output 2 Distribution Reference

Test case 7, the first row is LFNN captured outputs and the second row is FNN predicted outputs.

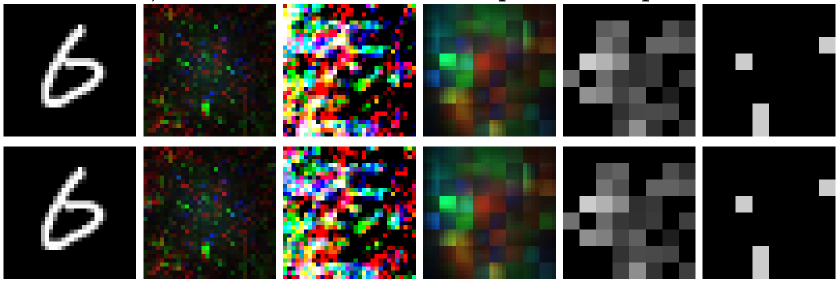

Input 1 Output 1 Input 2 Output 2 Distribution Reference

Test case 8, the first row is LFNN captured outputs and the second row is FNN predicted outputs.

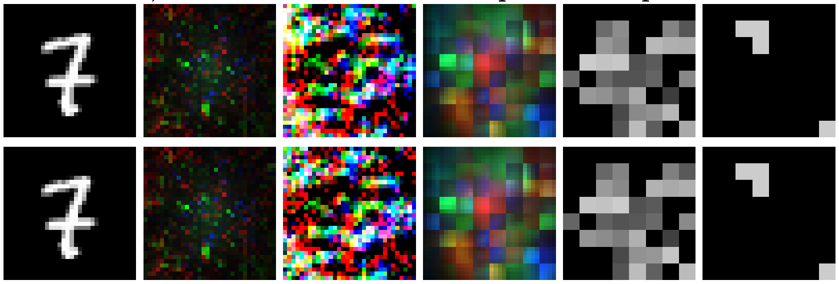

Input 1 Output 1 Input 2 Output 2 Distribution Reference

Test case 9, the first row is LFNN captured outputs and the second row is FNN predicted outputs.

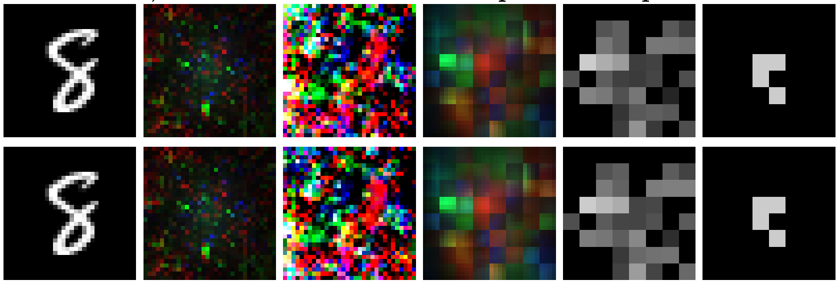

Input 1 Output 1 Input 2 Output 2 Distribution Reference

Test case 10, the first row is LFNN captured outputs and the second row is FNN predicted outputs.

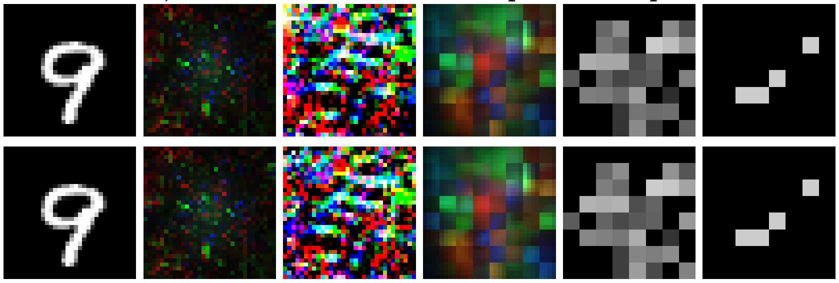

Input 1 Output 1 Input 2 Output 2 Distribution Reference

Figure S29. Layer outputs of MNIST Classification (2 Layer) for test cases 6 to 10.

S4.3 Results of CIFAR10 Classification

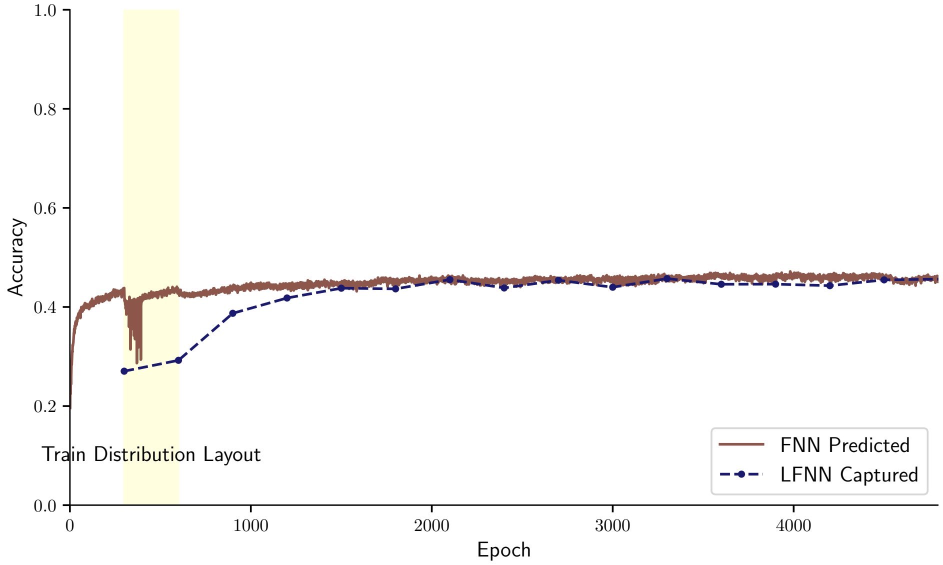

Figure S30. Evaluation of LFNN captured and FNN predicted results for CIFAR10 Classification.

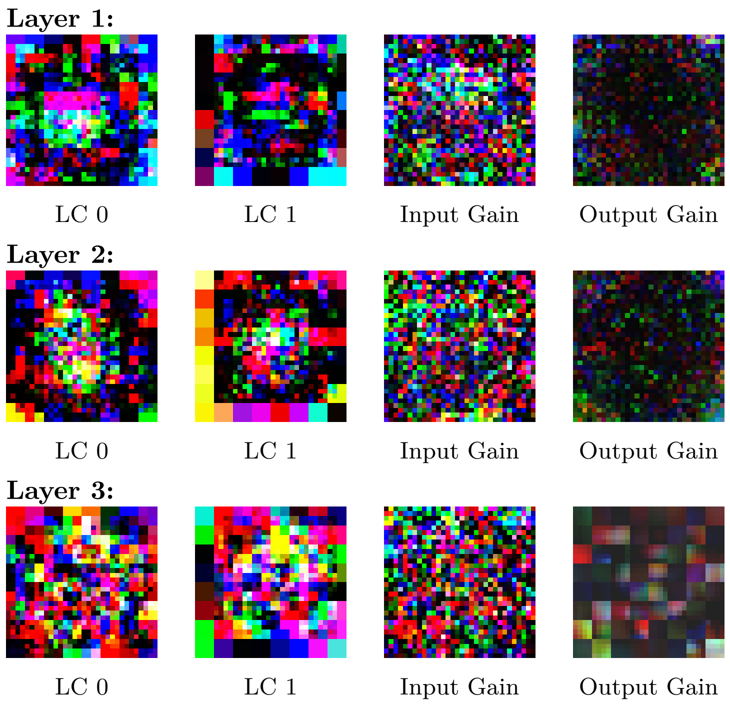

Figure S31. Hardware parameters of CIFAR10 Classification.

Test case 1, the first row is LFNN captured outputs and the second row is FNN predicted outputs.

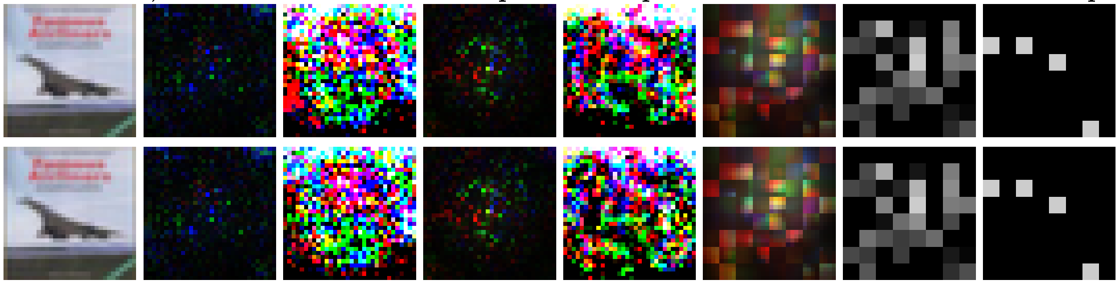

Input 1 Output 1 Input 2 Output 2 Input 3 Output 3 Distribution Reference

Test case 2, the first row is LFNN captured outputs and the second row is FNN predicted outputs.

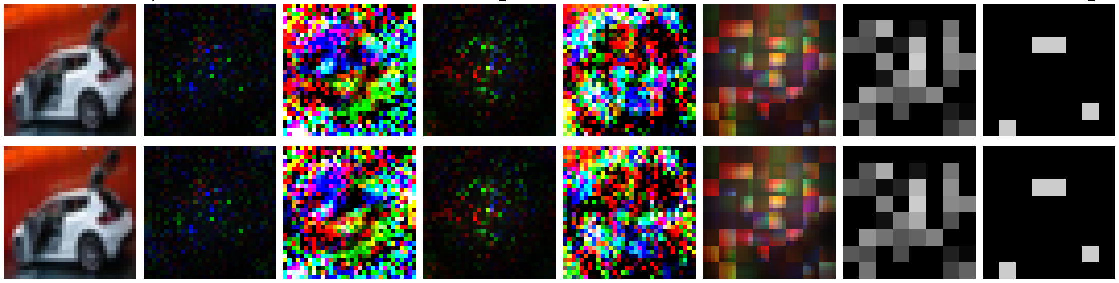

Input 1 Output 1 Input 2 Output 2 Input 3 Output 3 Distribution Reference

Test case 3, the first row is LFNN captured outputs and the second row is FNN predicted outputs.

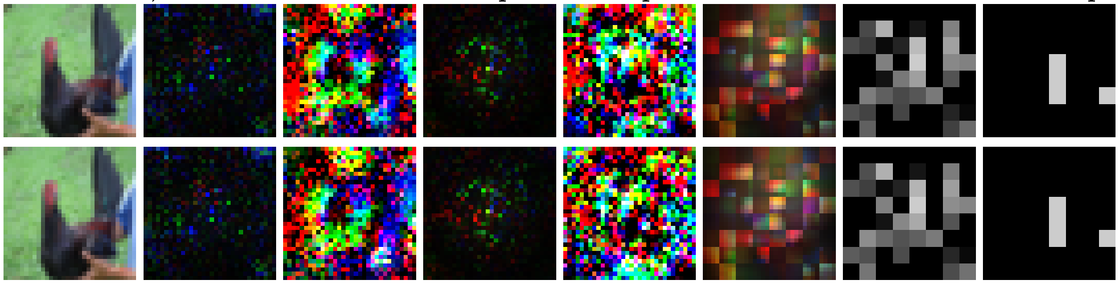

Input 1 Output 1 Input 2 Output 2 Input 3 Output 3 Distribution Reference

Test case 4, the first row is LFNN captured outputs and the second row is FNN predicted outputs.

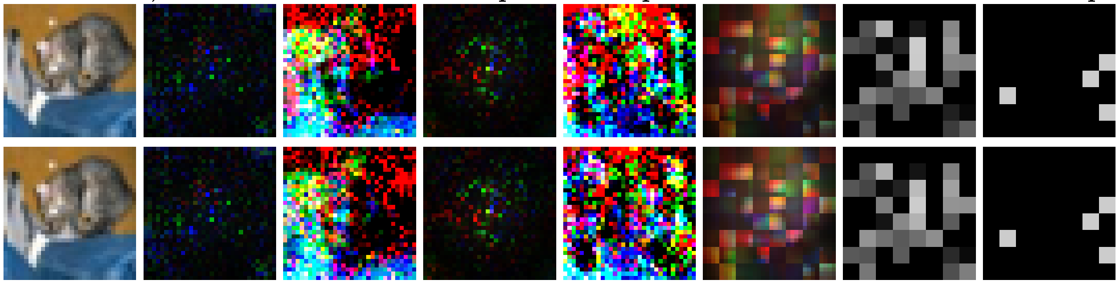

Input 1 Output 1 Input 2 Output 2 Input 3 Output 3 Distribution Reference

Test case 5, the first row is LFNN captured outputs and the second row is FNN predicted outputs.

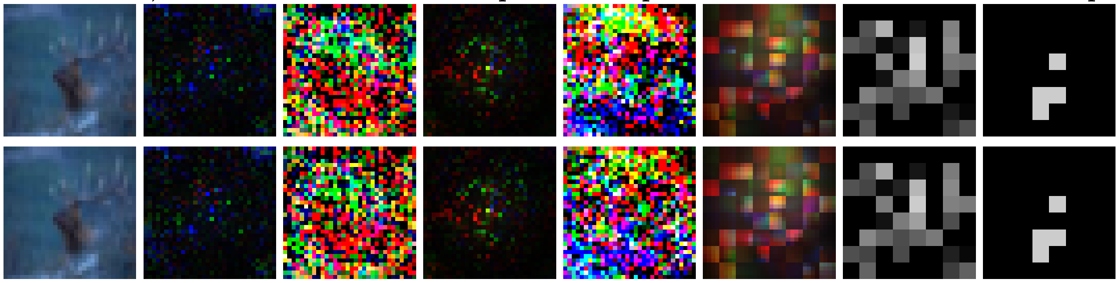

Input 1 Output 1 Input 2 Output 2 Input 3 Output 3 Distribution Reference

Figure S32. Layer outputs of CIFAR10 Classification for test cases 1 to 5.

Test case 6, the first row is LFNN captured outputs and the second row is FNN predicted outputs.

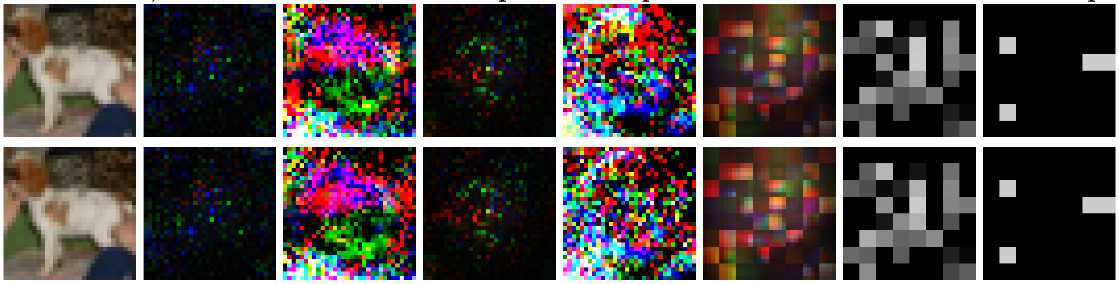

Test case 7, the first row is LFNN captured outputs and the second row is FNN predicted outputs.

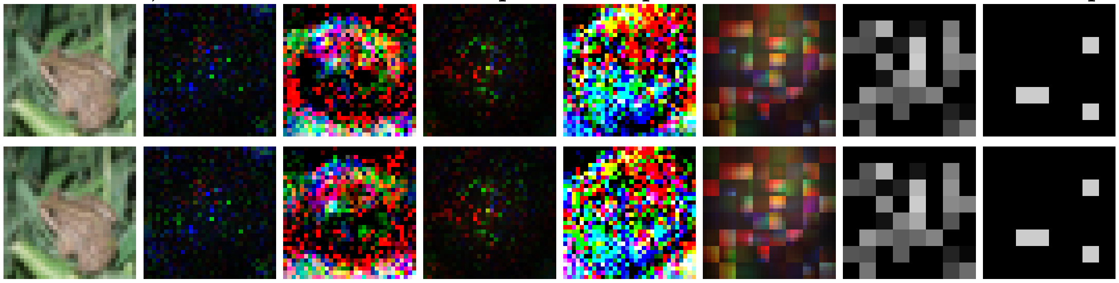

Test case 8, the first row is LFNN captured outputs and the second row is FNN predicted outputs.

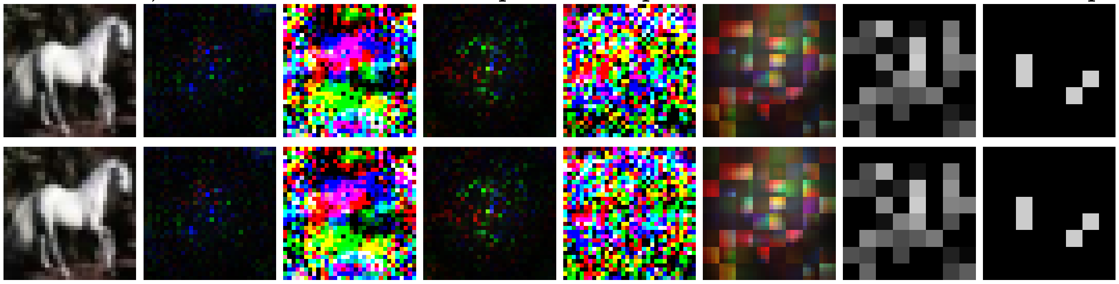

Test case 9, the first row is LFNN captured outputs and the second row is FNN predicted outputs.

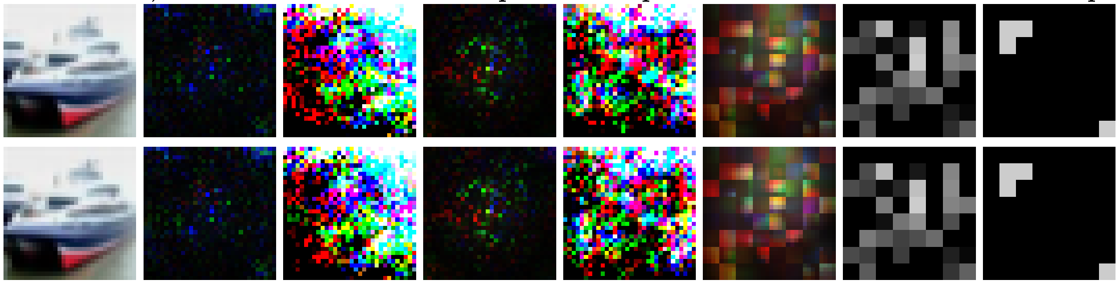

Test case 10, the first row is LFNN captured outputs and the second row is FNN predicted outputs.

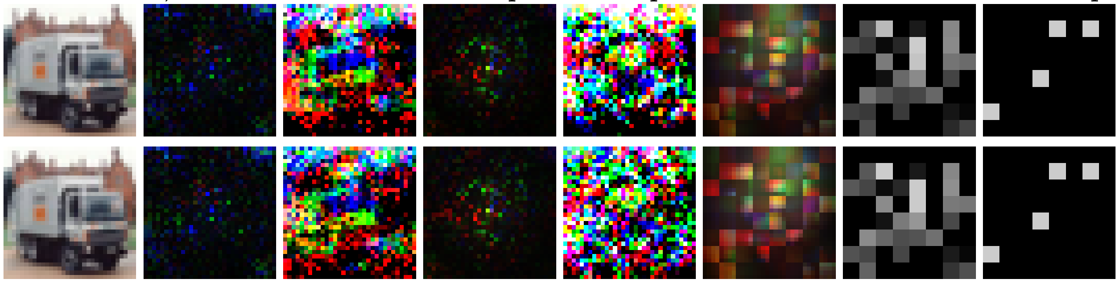

Figure S33. Layer outputs of CIFAR10 Classification for test cases 6 to 10.

S4.3.1 Result Analysis

As can be seen, the classification accuracy of the LFNN is lower in the CIFAR10 dataset than in the MNIST dataset. In the following sections, we analyze the possible reasons.

Deep learning has become a complicated system. It is supported by a highly active research society with many exciting discoveries every day, such as dataset generation, problem modeling, network architecture, loss design, training paradigm, and dedicated hardware. The final performance is jointly determined by all these factors, many of which are orthogonal to our study. These known factors affecting the performance of neural networks can be generally categorized into network complexity, network complexity, and learning complexity[10].

S4.3.2 Network Complexity

Network complexity partially depends on the hardware, including neuron complexity, the number of neurons in each layer, the number of layers, and the number and type of interconnection weights.

Neuron complexity could be viewed at aggregation function and activation function levels. At the activation function level, most digital neural networks use the ReLU activation layers. Because it is handy to follow this idea in our implementation for all experiments, the activation function level makes no difference between the CIFAR10 and MNIST datasets. At the aggregation function level, product and summation are two common functions of neural networks, which in our LFNN implementation are realized by the LC layers-based attenuation field. There are two physical limitations of the attenuation field compared with the vanilla digital neural network: the physically-limited modulation resolution and the lack of negative product operation. The lack of negative product operation is non-negligible, as discussed in Section S3.2. Nonetheless, it is irrelevant to the difference between the MNIST and CIFAR10 datasets since we use the same mechanism for both datasets. The modulation resolution is mainly determined by the number of trainable LC neurons at each neural network layer. As shown in Table S4, the number of LC neurons affects the performance in both datasets. While increasing the resolution gains 0.03% and 0.24% improvement in the MNIST dataset, the improvement in the CIFAR10 dataset is up to 2.08%. The possible explanation for this difference is that low modulation resolution cannot extract the detailed real-world image features in the CIFAR10 dataset. This could be the first reason for the decline of accuracy in the CIFAR10 dataset and can be alleviated by high-resolution LC panels.

Table S4. Accuracy comparison of different numbers of LC neurons with the numerical simulation. All neuron arrays are regularly aligned with two or three LC layers, and the LC layers equally split a spacing of 120 mm.

	**Regular-2**	**Regular-3**
**LC Neurons**	2048×3	3072×3
**1-Layer MNIST**	91.70%	91.73%
**2-Layer MNIST**	97.24%	97.51%
**3-Layer CIFAR10**	47.34%	49.42%

The number of neurons in each layer and **the number of layers** are viewed as hyperparameters of neural networks and significantly impact prediction accuracy. As far as we know, there is no systematic study that can provide theoretical guidance on how to choose optimal hyperparameters. Experimentally, the hyperparameters must match the complexity and size of the data. The network model might be too small for the data if the neuron number is too small, and the data might not be enough to train the model if the number of neurons is too large. Sometimes, the gradient might disappear if the number of neurons is too large, requiring sophisticated training techniques. Table S5 reports the accuracy with respect to these two hyperparameters in the CIFAR10 dataset. It is easy to see that increasing the number of neurons in each layer has no evident effects. On the other hand, while the digital DNN only slightly benefits from higher numbers of neural network layers, the LFNN simulation results become closer to the digital DNN. It shows that three layers might be enough for the CIFAR10 dataset using the digital DNN model. However, more neural network layers increase the modulation capability of the LFNN device, compensating for the insufficiency of modulation resolution and leading to closer performance of the digital DNN. In conclusion, training a dense LFNN device might be a promising way to increase the accuracy of complex datasets.

The number and type of interconnection weights determine the neuron's interconnection with previous layer neurons and the weight of interconnection (signal gain), which could be deterministic or fuzzy in nature. In our LFNN system, the number of interconnections is determined by the scattering angle of the input neuron and the distance between the input and output layers, and the weights of interconnection are partially affected by the layout of LC neurons. It is intuitive to see that if the scattered energy of an input neuron cannot cover the entire output layer, some interconnections would be reduced. Because the scattering angle is bounded by the

Table S5. Accuracy comparison of different numbers of neural network layers and neurons in each layer in the CIFAR10 dataset. The numbers in the brackets are the numbers of neurons in each layer. Note that it is not necessary that the higher number of neurons or layers, the better.

Layers	Digital DNN (3072)	LFNN Simulation (3072)	Digital DNN (6144)	LFNN Simulation (6144)
2	50.55	38.18%	50.31%	38.95%
3	53.62 %	49.42%	52.79%	49.53%
4	53.70 %	51.06%	52.85%	51.12%
5	53.81 %	51.59%	52.91%	51.71%

physical implementation of the LC panels (Figure S4), we conduct an experiment on various layer spacing (Table S1). The experiment results show that small (60 nm) layer spacing, i.e., fewer interconnections, produces a larger performance decline in the CIFAR10 dataset than the MNIST dataset. We believe it is because the subtle features of the CIFAR10 images demand connectivity between neurons on opposite angles. The performance gap between 120 nm and 240 nm becomes narrow but still exists, revealing a possibility to increase the LFNN's performance through such as adding some Fresnel lens. The weights of interconnection are determined by the LC neuron layout. Figure S1 and Table **??** show the impact of LC neuron layout on the performance. As can be seen, 3 LC planes and random neuron distribution yield higher prediction accuracy as they provide higher modulation resolution and computational flexibility, which can be utilized well by the following data-driven training process. In addition, results show that the CIFAR10 dataset benefits more than the MNIST dataset, and additional LC planes can continue to improve the accuracy.

S4.3.3 Problem Complexity

The performance does not depend only on the hardware and algorithm, but also on the problem complexity. In fact, the problem complexity is the primary reason for the different prediction accuracies between the CIFAR10 and MNIST datasets. It is common knowledge that the performance of deep-learning algorithms could vary significantly in different datasets. Take semantic segmentation as an example, the best records of mean Intersection over Union (IoU) on the PASCAL VOC 2012[11], Cityscapes[12], ADE20K[13], and DADA-seg[14] datasets are 90.5% ([15]), 85.2% ([16]), 61.4% ([17]), and 46.97% ([18]), respectively. The Cityscapes and DADA-seg are similar datasets that both focus on semantic understanding of urban street scenes, but the state-of-the-art (SOTA) performance gap is as high as 38.23%. Even using the same model, DeepLabV3+[19], the mean IoUs are 83.6% on the Cityscapes dataset, but only 26.8% DADA-seg dataset. Compared with the semantic segmentation task, the image classification task is a relatively well-studied task. However, the performance difference due to the problem complexity still exists. While the best record of top 1 accuracy has reached 91.0% ([20]) on the ImageNet dataset[21], the best top 1 accuracy is only 69.3% ([22]) on the Places205 dataset[23]. In the same model comparison, the recent MAE model[24] achieves 87.8% top 1 accuracy in the ImageNet dataset, but only 66.8% in the Places205 dataset. These publicly available data verifies that the problem complexity can be a dominant reason for the performance gap.

In our case, certain differences exist in the problem complexity of the CIFAR10 and the MNIST datasets. First, the **range of data** has a major role in the problem complexity. A pixel (or feature) with a large magnitude may appear to have more significance than the information it actually contains, and the network must learn to compensate for this inconsistency. While MNIST contains binarized digits, the images of CIFAR10 are 24bit RGB colors, producing a much larger value range and a more complex problem domain. Second, in our natural world, the **amount of information** of the problem determines the difficulty of understanding it. Unlike the simple 2D symbols in the MNIST dataset, CIFAR10 images of real-world objects contain information on shape, texture, posture, and lighting, making it harder to expose critical features for identification. Third, the **noise in the data** introduced by motion blurring, out-of-focus effects, and system noise reduces the signal-to-noise ratio of the CIFAR10 dataset. Forth, the **recognizability of classes** is fundamentally different between the two datasets. While the MNIST dataset contains symbols designed for easy recognization and reading, the CIFAR10 dataset contains naturally similar real-world objects and backgrounds with shared features and colors.

In our experiment, including the digital DNN, i.e., a standard multilayer perceptron (MLP), the numerical simulation, and the LFNN system all produce a very large accuracy gap between the CIFAR10 and MNIST datasets (Table S6). Therefore, we believe the primary reason for the difference is that the problem complexity of the CIFAR10 dataset has exceeded the capability of the standard densely connected neural network.

Table S6. Classification accuracy of four neuron array simulations, the actual LFNN captured output, the FNN predicted LFNN output, and the equal-layer digital dense neural network (DNN) reference. The LFNN has 12,288 trainable variables per layer, including 6,144 to control LC neurons and 6,144 to control the input/output intensity gains. The FNN has 28,438,144 trainable variables per layer. The digital dense neural network comprises dense layers connected by ReLU with neuron sizes of (784,10), (784,784,10), and (3072,3072,3072,10), respectively.

	**Regular-2**	**Regular-3**	**Normal-3**	**Uniform**	**LFNN**	**FNN**	**Digital DNN**
**LC Neurons**	2048×3	3072×3	3072×3	3072×3	2048×3		N.A.
**1-Layer MNIST**	91.03%	92.07%	92.40%	92.45%	91.02%	91.39%	92.71%
**2-Layer MNIST**	96.61%	97.30%	97.55%	97.65%	94.77%	95.45%	98.32%
**3-Layer CIFAR10**	47.48%	50.61%	51.73%	52.53%	45.62%	46.19%	53.62%

S4.3.4 Learning Complexity

The performance of neural networks depends upon training algorithms, selection of error functions, mode of error calculation, initialization of weights, and initialization of training parameters.

Training algorithms are critical for making full use of neural networks to learn datasets. Before the wide application of the back-propagation algorithm[25], there was no efficient way to train multi-layer deep neural networks. The development of back-propagation and other advanced training algorithms helped MLPs to achieve remarkable prediction accuracy over 99% in the MNIST dataset, the most popular benchmark in the early era of deep learning. Later, the CIFAR10 dataset was released as an open challenge for the classification of real-world objects, which is, unfortunately, too difficult for MLPs to achieve prediction accuracy higher than 60%[26]. Even using many recent advancements, including data augmentation, the accuracy does not exceed 70%[27]. In contrast, the prediction accuracy of convolutional neural networks (CNNs) easily surpassed 90% in the CIFAR10 dataset[28], which dispelled the doubts about neural network's performance in real-world data.

Even though CNNs show higher performance in visual tasks, there is no fundamental difference between the network architectures of CNNs and MLPs. In fact, CNNs can be viewed as a special subset of MLPs that neurons only interconnect their neighboring neurons of previous layers via shared weights. Because MLPs can represent arbitrary interconnections between neurons of adjacent layers, it is possible to embed a CNN into an MLP[29] or to utilize the idea of CNN in the LFNN. The fundamental difference between CNNs and MLPs is the training algorithms. CNNs are inspired by the biological connectivity pattern of neurons of the animal visual cortex. Individual neurons respond to stimuli only in a small receptive field, and the weights of connectivity are shared among same-layer neurons to ensure spatial invariance. These two features basically depend upon the training algorithm and are compatible with our LFNN design. However, there is no straightforward method to apply the training paradigm of CNN to the LFNN system. Because of the paradox between the spatial invariance requirement of CNNs and the spatial inconsistency of the hardware neurons, the weights cannot be directly shared between neurons. It is a challenging but promising future work to introduce the biologically inspired connectivity pattern to our functional learning paradigm to compensate for this paradox, which could significantly improve the system's performance in real-world inputs.

Selection of error functions also affects the training results. The cross-entropy loss is a common choice for image classification. Other widely used losses include L1, L2, mean absolute error, mean squared error, and so on. Besides, advanced perceptual losses have been reported to be more robust in capturing real-world objects and perceptual features[30, 31]. Future explorations in the error functions might enhance the accuracy of real-world image datasets like CIFAR10.

Mode of error calculation is the pattern of updating weights. Commonly, the error can be calculated after presenting each input or after a batch of inputs, producing case-dependent results. In our functional learning paradigm, the combination of P-learning and Z-learning enables many possible error calculation modes. It requires case-by-case research to study these possibilities in different datasets for production-ready deployments. This can trigger many follow-on research.

Initialization of weights and training parameters are also important hyperparameters that can improve the performance via careful tuning. However, they are not case-dependent and thus not the primary reason for the accuracy gap since we use the same parameters to train the CIFAR10 and MNIST datasets.

S4.3.5 Summary

In conclusion, the performance of neural networks involves various factors like hardware, algorithm, and data complexity. It is a wide-ranging and active ongoing study to improve the overall performance of neural networks,

especially in the case of complicated real-world inputs. Simple problems like the MNIST dataset were first well addressed, followed by the challenging real-world CIFAR10 datasets, and finally, more difficult problems containing high dynamicity and complicated lighting. The step-by-step process conforms to human intuition and scientific law. Similar to MLPs, our LFNN system and FL learning paradigm have gained promising results. In fact, our performance, even though achieved in an incoherent system, is on par with those of coherent optical neural networks. While there is a performance decline in the CIFAR10 dataset, it is not surprising, considering the much higher problem complexity, which affects all kinds of neural networks. We believe it is not a systematic problem of optical neural networks, instead can be resolved by introducing ideas from CNNs and other recent studies. While the evolution from MLPs to CNNs takes decades in history, it is promising to see a much faster advance of the optical neural network, given many successful explorations of digital neural networks.

S4.4 Results of Digit Recognition

Table S7. Digit recognition accuracy using 1-layer LFNN.

**Digit**	0	1	2	3	4	5	6	7	8	9
**Accuracy**	97.88%	98.05%	96.13%	94.16%	95.64%	94.08%	96.03%	96.02%	91.93%	92.86%

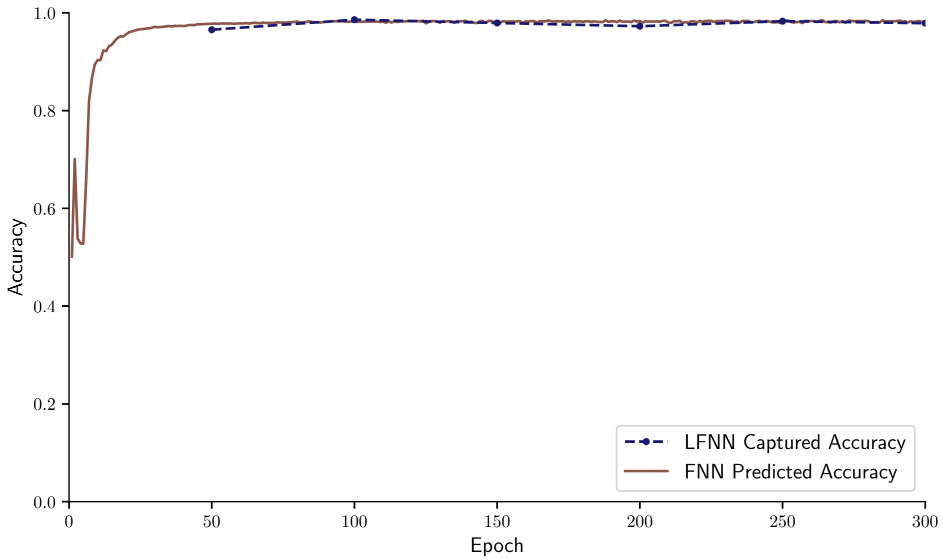

Figure S34. Evaluation of LFNN captured and FNN predicted results for Digit 0 Recognition.

Layer 1:

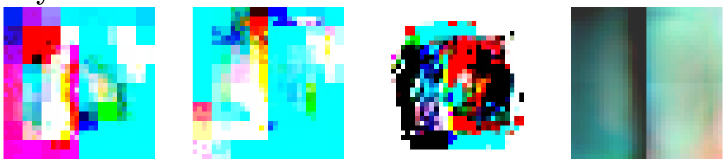

LC 0 | LC 1 | Input Gain | Output Gain

Figure S35. Hardware parameters of Digit 0 Recognition.

Test case 1, the first row is LFNN captured outputs and the second row is FNN predicted outputs.

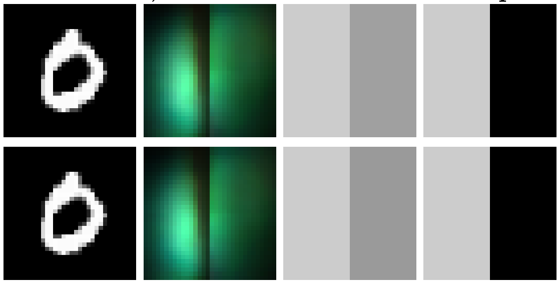

Input 1 Output 1 Distribution Reference

Test case 2, the first row is LFNN captured outputs and the second row is FNN predicted outputs.

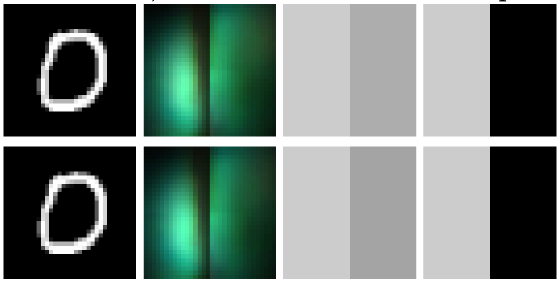

Input 1 Output 1 Distribution Reference

Test case 3, the first row is LFNN captured outputs and the second row is FNN predicted outputs.

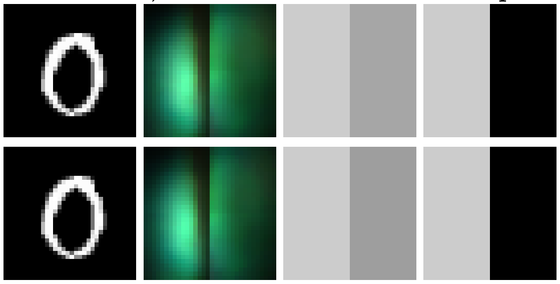

Input 1 Output 1 Distribution Reference

Test case 4, the first row is LFNN captured outputs and the second row is FNN predicted outputs.

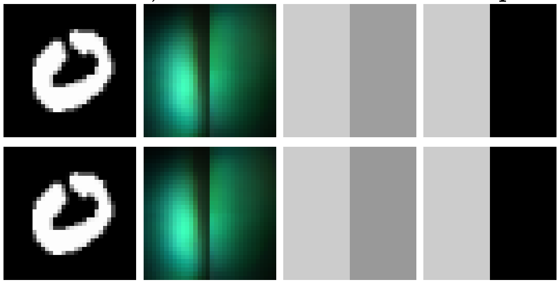

Input 1 Output 1 Distribution Reference

Test case 5, the first row is LFNN captured outputs and the second row is FNN predicted outputs.

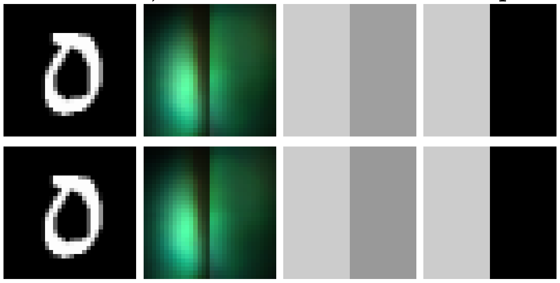

Input 1 Output 1 Distribution Reference

Figure S36. Layer outputs of Digit 0 Recognition for test cases 1 to 5.

Test case 6, the first row is LFNN captured outputs and the second row is FNN predicted outputs.

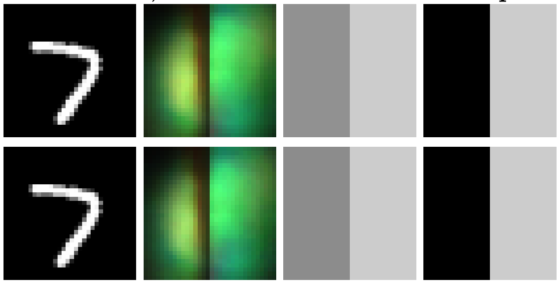

Input 1 Output 1 Distribution Reference

Test case 7, the first row is LFNN captured outputs and the second row is FNN predicted outputs.

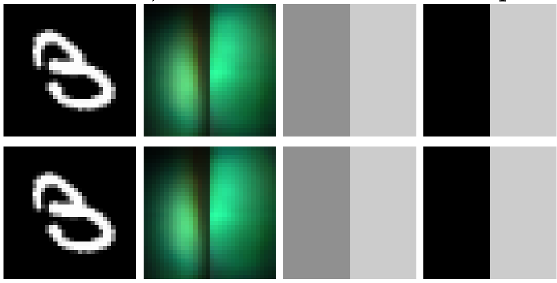

Input 1 Output 1 Distribution Reference

Test case 8, the first row is LFNN captured outputs and the second row is FNN predicted outputs.

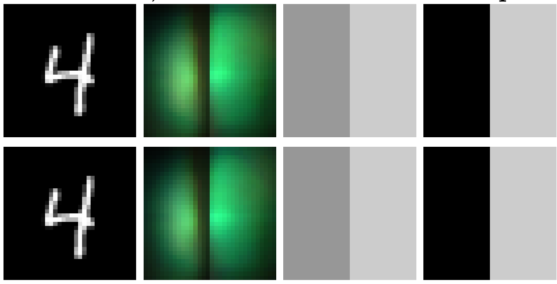

Input 1 Output 1 Distribution Reference

Test case 9, the first row is LFNN captured outputs and the second row is FNN predicted outputs.

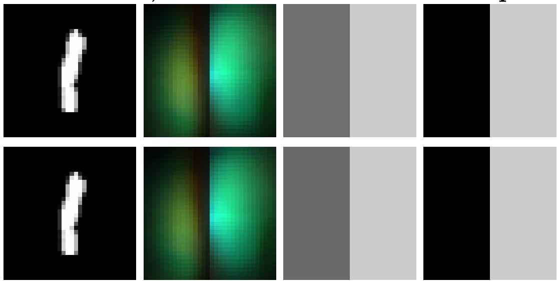

Input 1 Output 1 Distribution Reference

Test case 10, the first row is LFNN captured outputs and the second row is FNN predicted outputs.

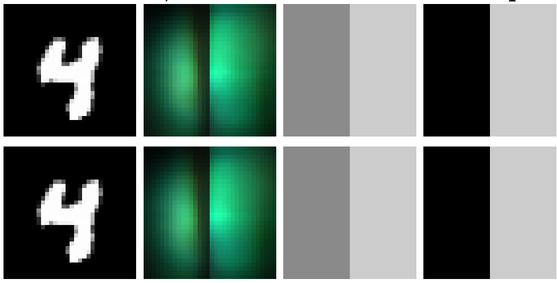

Input 1 Output 1 Distribution Reference

Figure S37. Layer outputs of Digit 0 Recognition for test cases 6 to 10.

S4.5 Results of Object Recognition

Table S8. Object recognition accuracy using 1-layer LFNN.

**Object**	Airplane	Automobile	Bird	Cat	Deer	Dog	Frog	Horse	Ship	Truck
**Accuracy**	76.88%	77.95%	63.93%	64.37%	71.61%	66.98%	78.23%	72.40%	79.02%	76.38%

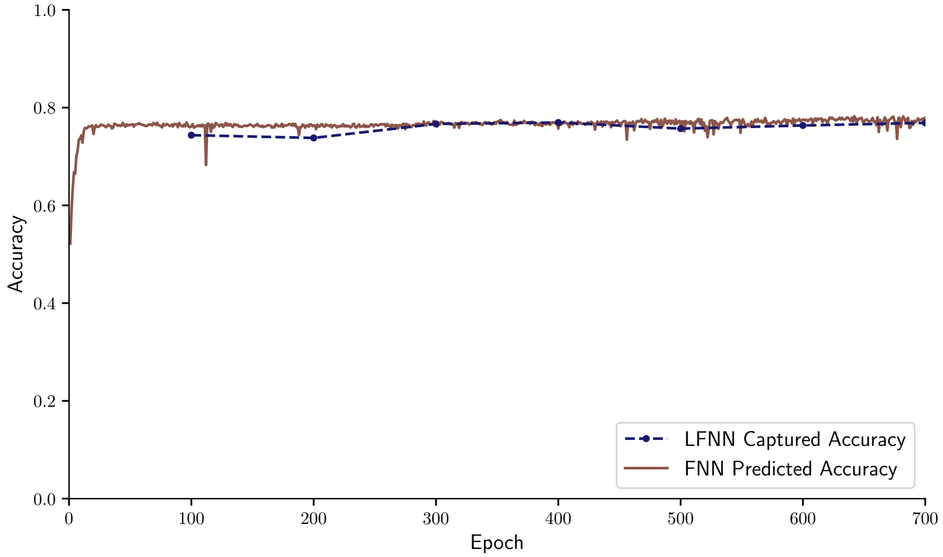

Figure S38. Evaluation of LFNN captured and FNN predicted results for Plane Recognition.

Layer 1:

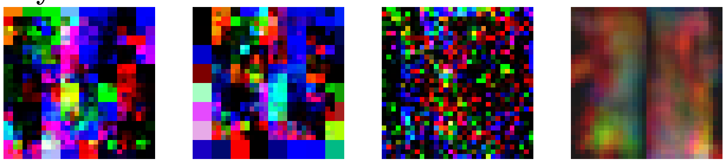

LC 0   LC 1   Input Gain   Output Gain

Figure S39. Hardware parameters of Plane Recognition.

Test case 1, the first row is LFNN captured outputs and the second row is FNN predicted outputs.

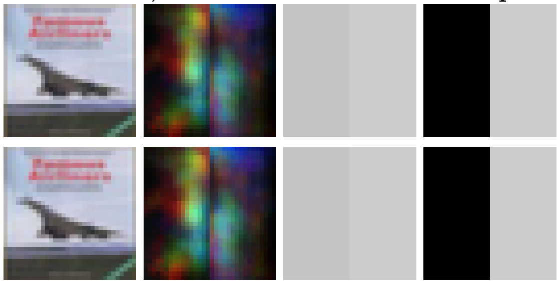

Input 1 Output 1 Distribution Reference

Test case 2, the first row is LFNN captured outputs and the second row is FNN predicted outputs.

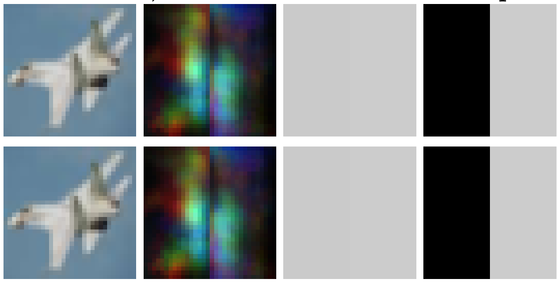

Input 1 Output 1 Distribution Reference

Test case 3, the first row is LFNN captured outputs and the second row is FNN predicted outputs.

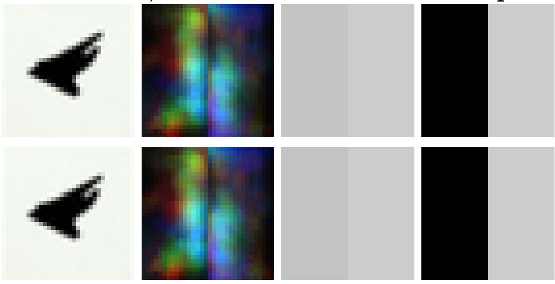

Input 1 Output 1 Distribution Reference

Test case 4, the first row is LFNN captured outputs and the second row is FNN predicted outputs.

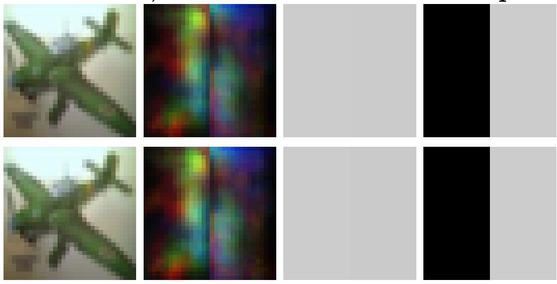

Input 1 Output 1 Distribution Reference

Test case 5, the first row is LFNN captured outputs and the second row is FNN predicted outputs.

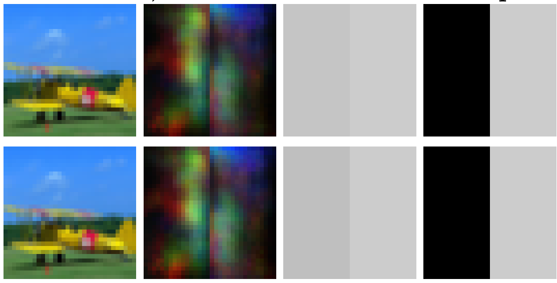

Input 1 Output 1 Distribution Reference

Figure S40. Layer outputs of Plane Recognition for test cases 1 to 5.

Test case 6, the first row is LFNN captured outputs and the second row is FNN predicted outputs.

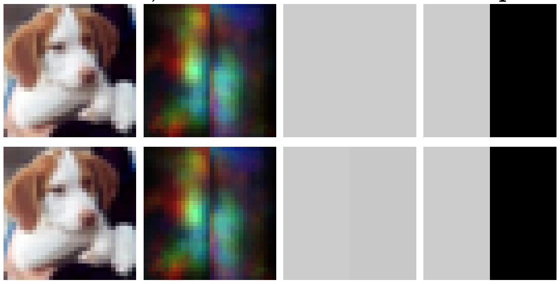

Input 1 Output 1 Distribution Reference

Test case 7, the first row is LFNN captured outputs and the second row is FNN predicted outputs.

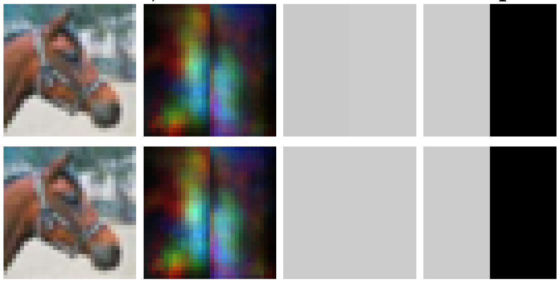

Input 1 Output 1 Distribution Reference

Test case 8, the first row is LFNN captured outputs and the second row is FNN predicted outputs.

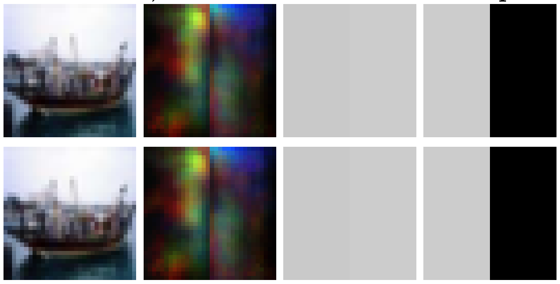

Input 1 Output 1 Distribution Reference

Test case 9, the first row is LFNN captured outputs and the second row is FNN predicted outputs.

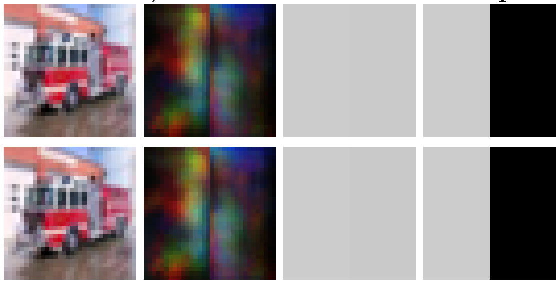

Input 1 Output 1 Distribution Reference

Test case 10, the first row is LFNN captured outputs and the second row is FNN predicted outputs.

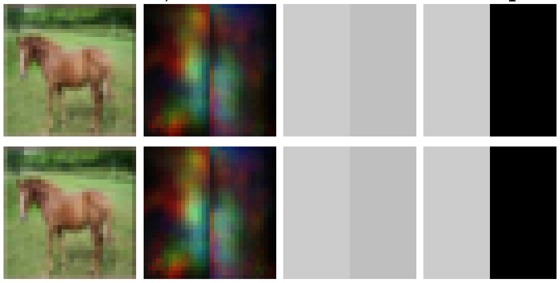

Input 1 Output 1 Distribution Reference

Figure S41. Layer outputs of Plane Recognition for test cases 6 to 10.

S4.6 Results of Depth Estimation

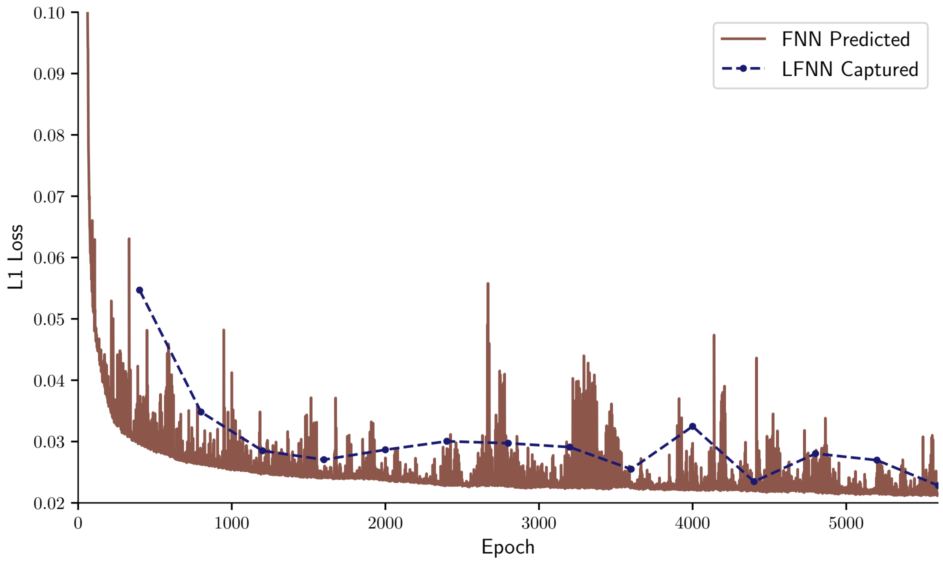

Figure S42. Evaluation of LFNN captured and FNN predicted results for Depth Estimation.

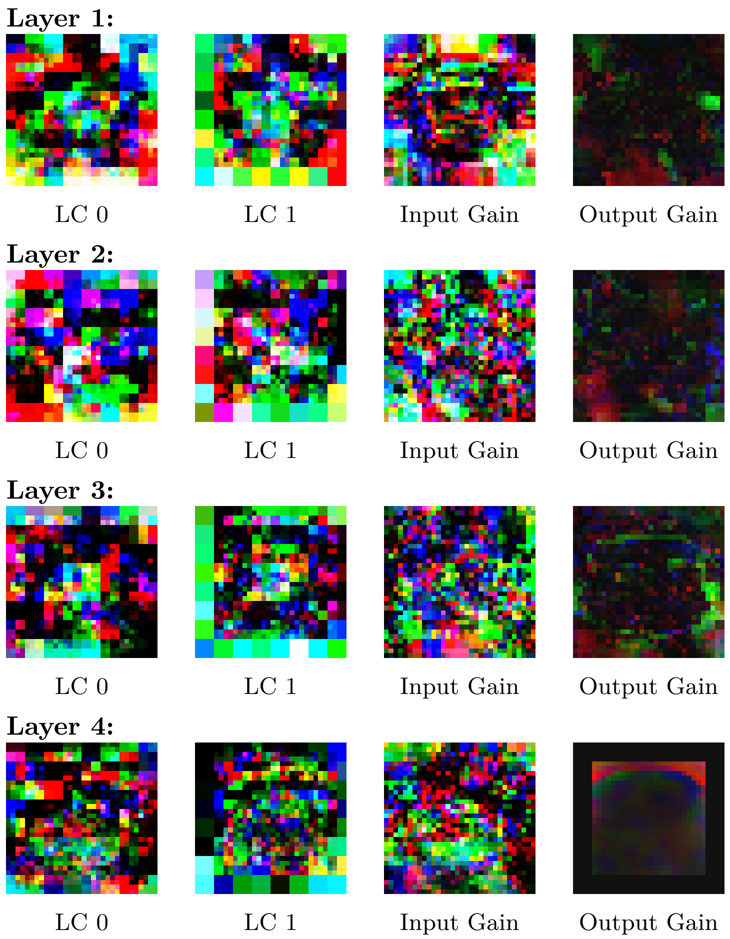

Figure S43. Hardware parameters of Depth Estimation.

Test case 1, the first row is LFNN captured outputs and the second row is FNN predicted outputs.

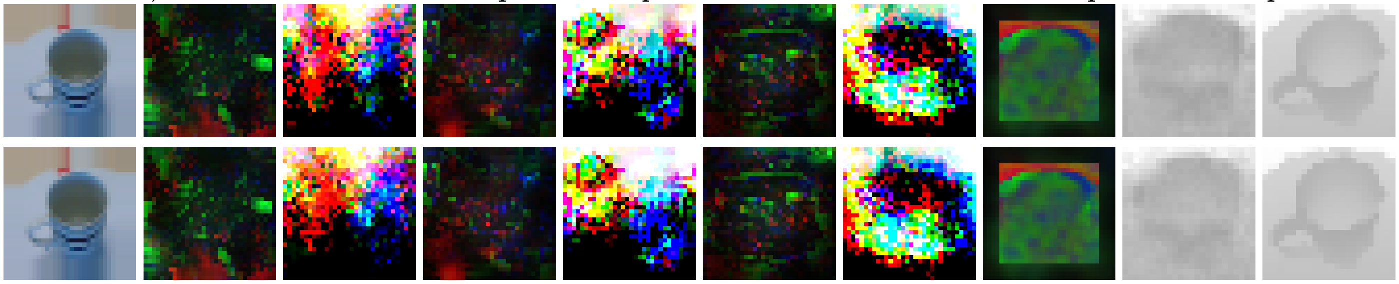

Input 1 Output 1 Input 2 Output 2 Input 3 Output 3 Input 4 Output 4 Depth Reference

Test case 2, the first row is LFNN captured outputs and the second row is FNN predicted outputs.

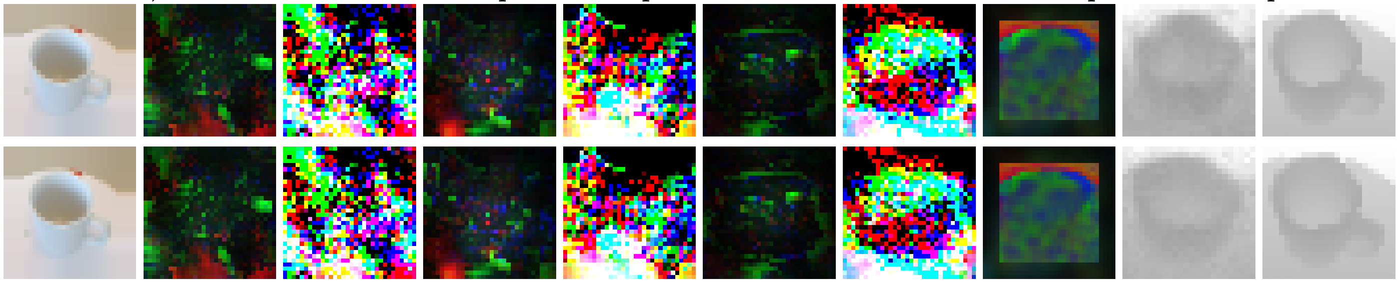

Input 1 Output 1 Input 2 Output 2 Input 3 Output 3 Input 4 Output 4 Depth Reference

Test case 3, the first row is LFNN captured outputs and the second row is FNN predicted outputs.

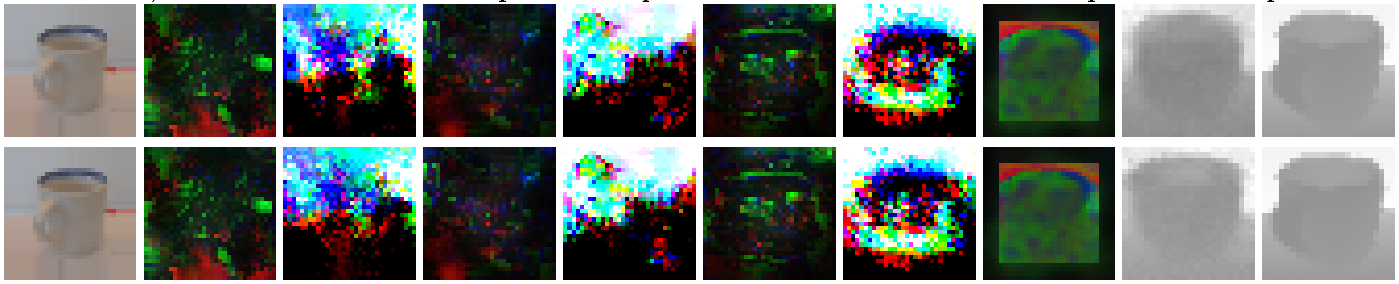

Input 1 Output 1 Input 2 Output 2 Input 3 Output 3 Input 4 Output 4 Depth Reference

Test case 4, the first row is LFNN captured outputs and the second row is FNN predicted outputs.

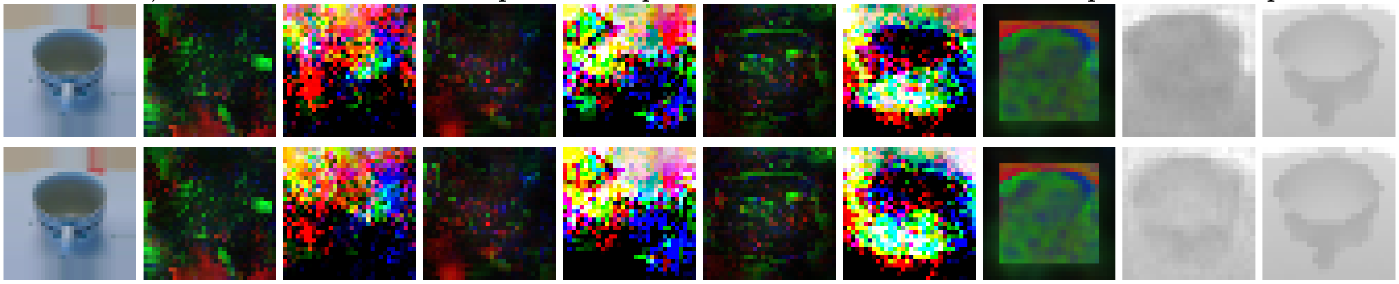

Input 1 Output 1 Input 2 Output 2 Input 3 Output 3 Input 4 Output 4 Depth Reference

Test case 5, the first row is LFNN captured outputs and the second row is FNN predicted outputs.

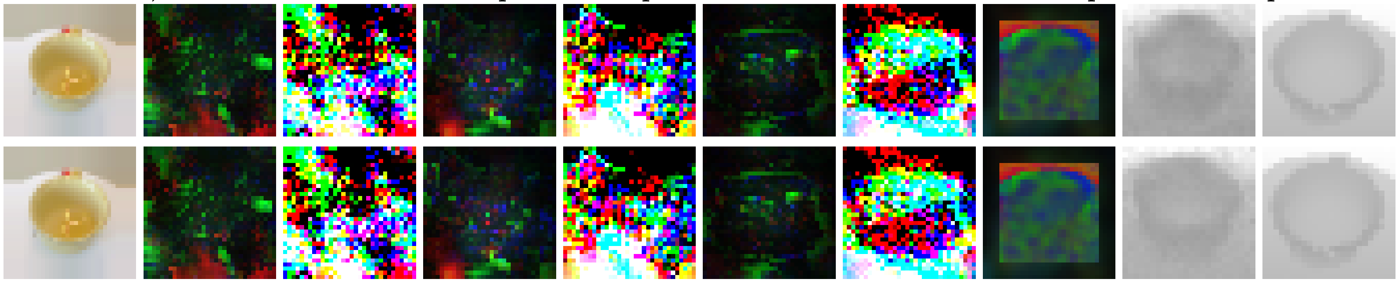

Input 1 Output 1 Input 2 Output 2 Input 3 Output 3 Input 4 Output 4 Depth Reference

Figure S44. Layer outputs of Depth Estimation for test cases 1 to 5.

Test case 6, the first row is LFNN captured outputs and the second row is FNN predicted outputs.

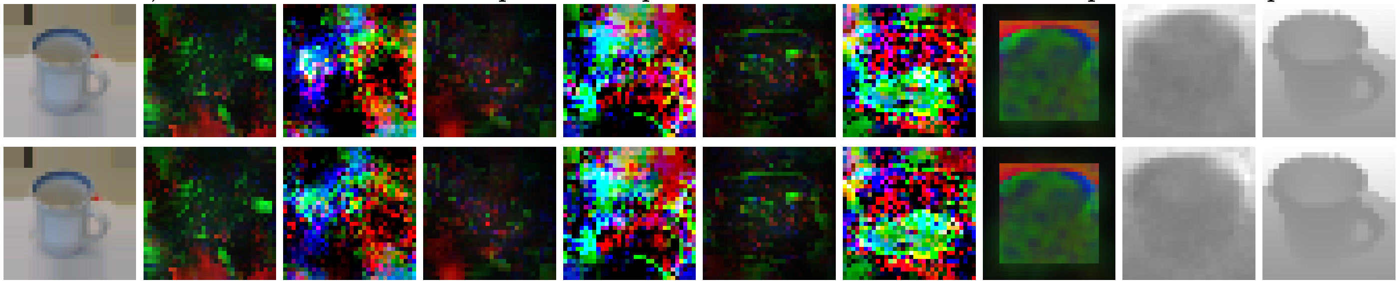

Input 1 | Output 1 | Input 2 | Output 2 | Input 3 | Output 3 | Input 4 | Output 4 | Depth | Reference

Test case 7, the first row is LFNN captured outputs and the second row is FNN predicted outputs.

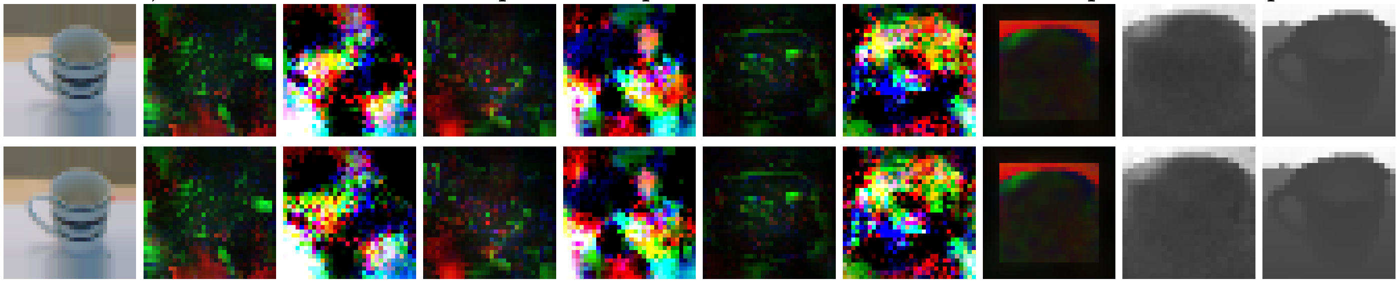

Input 1 | Output 1 | Input 2 | Output 2 | Input 3 | Output 3 | Input 4 | Output 4 | Depth | Reference

Test case 8, the first row is LFNN captured outputs and the second row is FNN predicted outputs.

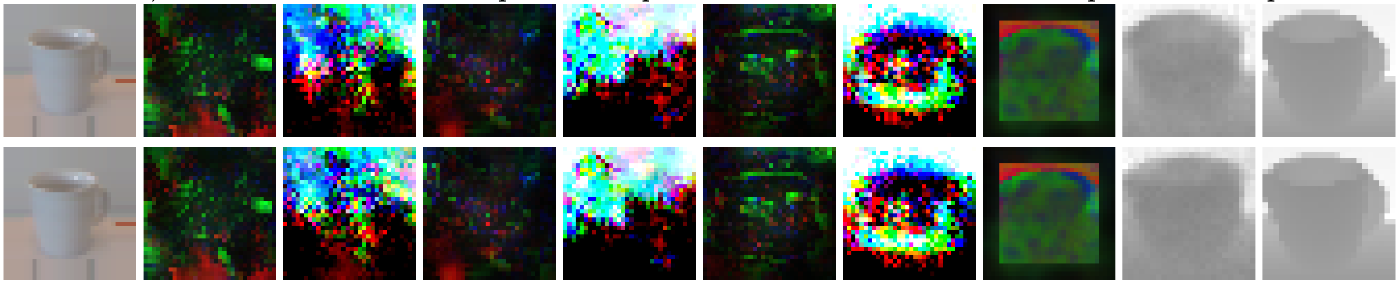

Input 1 | Output 1 | Input 2 | Output 2 | Input 3 | Output 3 | Input 4 | Output 4 | Depth | Reference

Test case 9, the first row is LFNN captured outputs and the second row is FNN predicted outputs.

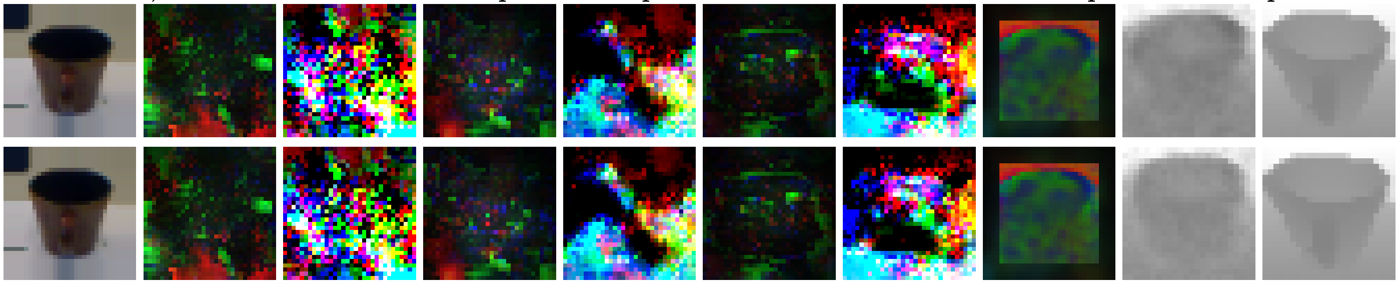

Input 1 | Output 1 | Input 2 | Output 2 | Input 3 | Output 3 | Input 4 | Output 4 | Depth | Reference

Test case 10, the first row is LFNN captured outputs and the second row is FNN predicted outputs.

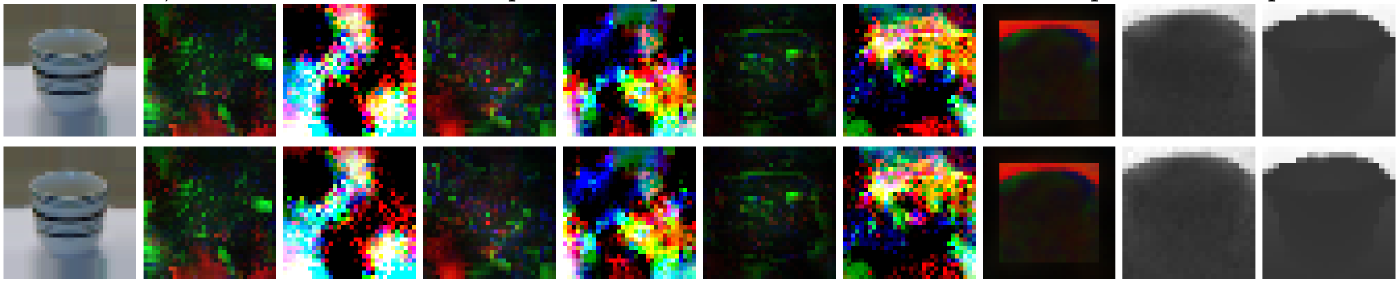

Input 1 | Output 1 | Input 2 | Output 2 | Input 3 | Output 3 | Input 4 | Output 4 | Depth | Reference

Figure S45. Layer outputs of Depth Estimation for test cases 6 to 10.

References

1. Wetzstein, G., Lanman, D., Hirsch, M. & Raskar, R. Tensor Displays: Compressive Light Field Synthesis using Multilayer Displays with Directional Backlighting. *ACM Trans. Graph. (Proc. SIGGRAPH)* **31**, 1–11 (2012).

2. Lin, X. *et al.* All-optical machine learning using diffractive deep neural networks. *Science* **361**, 1004–1008 (2018).

3. Mengu, D. *et al.* Misalignment resilient diffractive optical networks. *Nanophotonics* **9**, 4207–4219 (2020).

4. Shen, Y. *et al.* Deep learning with coherent nanophotonic circuits. *Nat. Photonics* **11**, 441 (2017).

5. Zhang, H. *et al.* Efficient on-chip training of optical neural networks using genetic algorithm. *ACS Photonics* (2021).

6. LeCun, Y. & Cortes, C. MNIST handwritten digit database. http://yann.lecun.com/exdb/mnist/ (2010).

7. Krizhevsky, A. Learning multiple layers of features from tiny images. *Univ. Tor.* (2009).

8. Lai, K., Bo, L., Ren, X. & Fox, D. A large-scale hierarchical multi-view rgb-d object dataset. In *2011 IEEE international conference on robotics and automation*, 1817–1824 (IEEE, 2011).

9. Kingma, D. P. & Ba, J. Adam: A method for stochastic optimization. *arXiv preprint arXiv:1412.6980* (2014).

10. Chaturvedi, D. K. Soft computing. *Stud. Comput. Intell.* **103**, 51–85 (2008).

11. Everingham, M. *et al.* The pascal visual object classes challenge: A retrospective. *Int. journal computer vision* **111**, 98–136 (2015).

12. Cordts, M. *et al.* The cityscapes dataset for semantic urban scene understanding. In *Proceedings of the IEEE conference on computer vision and pattern recognition*, 3213–3223 (2016).

13. Zhou, B. *et al.* Scene parsing through ade20k dataset. In *Proceedings of the IEEE conference on computer vision and pattern recognition*, 633–641 (2017).

14. Zhang, J., Yang, K. & Stiefelhagen, R. Exploring event-driven dynamic context for accident scene segmentation. *IEEE Transactions on Intell. Transp. Syst.* **23**, 2606–2622 (2021).

15. Zoph, B. *et al.* Rethinking pre-training and self-training. *Adv. neural information processing systems* **33**, 3833–3845 (2020).

16. Chen, Z. *et al.* Vision transformer adapter for dense predictions. *arXiv preprint arXiv:2205.08534* (2022).

17. Wei, Y. *et al.* Contrastive learning rivals masked image modeling in fine-tuning via feature distillation. *arXiv preprint arXiv:2205.14141* (2022).

18. Luo, X. *et al.* Towards robust semantic segmentation of accident scenes via multi-source mixed sampling and meta-learning. In *Proceedings of the IEEE/CVF Conference on Computer Vision and Pattern Recognition*, 4429–4439 (2022).

19. Chen, L.-C., Zhu, Y., Papandreou, G., Schroff, F. & Adam, H. Encoder-decoder with atrous separable convolution for semantic image segmentation. In *Proceedings of the European conference on computer vision (ECCV)*, 801–818 (2018).

20. Yu, J. *et al.* Coca: Contrastive captioners are image-text foundation models. *arXiv preprint arXiv:2205.01917* (2022).

21. Deng, J. *et al.* Imagenet: A large-scale hierarchical image database. In *2009 IEEE conference on computer vision and pattern recognition*, 248–255 (Ieee, 2009).

22. Liu, J., Huang, X., Liu, Y. & Li, H. Mixmim: Mixed and masked image modeling for efficient visual representation learning. *arXiv preprint arXiv:2205.13137* (2022).

23. Zhou, B., Lapedriza, A., Xiao, J., Torralba, A. & Oliva, A. Learning deep features for scene recognition using places database. *Adv. neural information processing systems* **27** (2014).

24. He, K. *et al.* Masked autoencoders are scalable vision learners. In *Proceedings of the IEEE/CVF Conference on Computer Vision and Pattern Recognition*, 16000–16009 (2022).

25. Rumelhart, D. E., Hinton, G. E. & Williams, R. J. Learning internal representations by error propagation. Tech. Rep., California Univ San Diego La Jolla Inst for Cognitive Science (1985).

26. Raiko, T., Valpola, H. & LeCun, Y. Deep learning made easier by linear transformations in perceptrons. In *Artificial intelligence and statistics*, 924–932 (PMLR, 2012).

27. Lin, Z., Memisevic, R. & Konda, K. How far can we go without convolution: Improving fully-connected networks. *arXiv preprint arXiv:1511.02580* (2015).

28. Lee, C.-Y., Xie, S., Gallagher, P., Zhang, Z. & Tu, Z. Deeply-supervised nets. In *Artificial intelligence and statistics*, 562–570 (PMLR, 2015).

29. Tolstikhin, I. O. *et al.* Mlp-mixer: An all-mlp architecture for vision. *Adv. Neural Inf. Process. Syst.* **34**, 24261–24272 (2021).

30. Johnson, J., Alahi, A. & Fei-Fei, L. Perceptual losses for real-time style transfer and super-resolution. In *European conference on computer vision*, 694–711 (Springer, 2016).

31. Zhang, R., Isola, P., Efros, A. A., Shechtman, E. & Wang, O. The unreasonable effectiveness of deep features as a perceptual metric. In *Proceedings of the IEEE conference on computer vision and pattern recognition*, 586–595 (2018).
